# Covalent-Assembly Based Fluorescent Probes for Detection of hNQO1 and Imaging in Living Cells

**DOI:** 10.3389/fchem.2020.00756

**Published:** 2020-08-26

**Authors:** Jialing Han, Longhao Cheng, Ya Zhu, Xiaowei Xu, Chaoliang Ge

**Affiliations:** ^1^Department of Pharmacy, The First Affiliated Hospital of Anhui Medical University, Hefei, China; ^2^Haimen People's Hospital, Nantong, China; ^3^School of Pharmacy, China Pharmaceutical University, Nanjing, China

**Keywords:** NQO1, fluorescent probe, cell imaging, covalent assembly, living cell

## Abstract

Human NAD(P)H: quinone oxidoreductase (hNQO1) is an important biomarker for human malignant tumors. Detection of NQO1 accurately is of great significance to improve the early diagnosis of cancer and prognosis of cancer patients. In this study, based on the covalent assembly strategy, hNQO1-activated fluorescent probes **1** and **2** are constructed by introducing coumarin precursor 2-cyano-3-(4-(diethylamino)-2-hydroxyphenyl) acrylic acid and self-immolative linkers. Under reaction with hNQO1 and NADH, turn-on fluorescence appears due to *in-situ* formation of the organic fluorescent compound 7-diethylamino-3-cyanocoumarin, and fluorescent intensity changes significantly. Probe **1** and **2** for detection of hNQO1 are not interfered by other substances and have low toxicity in cells. In addition to quantitative detection of hNQO1 *in vitro*, they have also been successfully applied to fluorescent imaging in living cells.

## Introduction

The presence of overexpressed reductase in solid tumor cells is the key to studying cancer progression. Rapid and selective reporting of cancer-related enzymes and other biologically active substances in cells will enable people to better understand tumor progression and invasiveness at the molecular level and methods of cancer diagnosis and treatment based on precision medicine. It also helps to study the targeted activation of chemotherapy drugs in tumors (Blum et al., 2007).

NAD(P)H: quinone oxidoreductase 1 (NQO1) is a cytoplasmic flavin protease that is widely distributed in the human body and can catalyze a two-electron reduction of various quinones to hydroquinone in a manner dependent on NAD(P)H (Ernster, 1967; Zhu et al., 2007; Bianchet et al., 2008; Siegel et al., 2012; Ross and Siegel, 2017; Beaver et al., 2019). Depending on the chemical properties of the hydroquinone formed, the reduction process may be a detoxification process or a biological activation process. In addition to participating in the detoxification metabolism of exogenous toxic substances in human body (Bianchet et al., 2008), NQO1 has also been found to participate in other biological processes, such as antioxidant function (Wefers et al., 1984), superoxide scavenging (Siegel et al., 2004), and regulation of the stability of specific proteins under oxidative stress (Nioi and Hayes, 2004; Dinkova-Kostova and Talalay, 2010). In many human solid tumors, the expression level is 5–200 times that of normal tissues (Zhang et al., 2018). For example, the expression of NQO1 in the liver of normal people is not abundant, but the expression level of NQO1 in the liver cancer is significantly higher than that of normal tissues by nearly 50 times (Aleksunes et al., 2006; Rougée et al., 2016). NQO1 is also highly expressed in other tumor cells, such as breast cancer (Marin et al., 1997; Yang et al., 2014), lung cancer (Siegel et al., 1998; Bey et al., 2007; Li et al., 2015), prostate cancer (Dong et al., 2010), gastric cancer (Lin et al., 2014), colon cancer (Ji et al., 2014), pancreatic cancer (Ough et al., 2005), head and neck cancer (Li et al., 2016), and cholangiocarcinoma (Wakai et al., 2011). In addition, high expression of NQO1 is closely related to the occurrence and development of cancer, tumor invasion, resistance to chemotherapy, and poor prognosis of patients.

To assess the activity of hNQO1 in living tumor model accurately and to distinguish metastasis in healthy tissue based on fluorescent methods immediately, we need to develop enzyme-activated fluorescent probes that respond quickly and have high sensitivity (Frangioni, 2008; Lee et al., 2010; Zhang J. J. et al., 2019). This type of probe can not only perform non-invasive detection on patients, but also keep normal cells intact. Real-time fluorescent imaging can achieve the purpose of constant monitoring and diagnosis. A new design strategy, the principle of covalent assembly, is used in the design and application of probes (Wu and Anslyn, 2005; Peng et al., 2012; Park and Kim, 2013; Quesneau et al., 2019). This strategy is mainly based on the reaction of non-fluorescent compounds triggered or catalyzed by the analyte, and then releases oxygen anions, which then attack the carbonyl carbon in the molecule to undergo a lactonization reaction, thereby forming a fluorescent group *in situ*. The advantage of this strategy is that it can greatly reduce the signal generated by the fluorescent probe itself and improve the target-to-background ratio.

Recently, a variety of NQO1-specific fluorescent probes have been reported (Punganuru et al., 2018; Kong et al., 2019; Park et al., 2019; Yuan et al., 2019; Zhang C. Y. et al., 2019; Zhu et al., 2019), but few of these probes were designed based on the principle of covalent assembly. Therefore, in order to improve the target-to-background ratio of the fluorescent probe, we adopted the principle of covalent assembly and designed NQO1-activited fluorescent probes **1** and **2** with trimethyl-locked quinone propionic acid (Q_3_PA) as the recognition group, coumarin precursor 2-cyano-3-(4-(diethylamino) 2-Hydroxyphenyl) acrylic acid as the fluorescent signal group and p-hydroxybenzyl alcohol or 2-mercaptoethanol as the spontaneously cleavable linker. These two probes not only have high selectivity and sensitivity against NQO1, but also have been successfully used in intracellular imaging, showing good potential in cancer diagnosis.

## Materials and Methods

### Instruments and Materials

^1^H NMR was recorded on Bruker AV-300 (300 MHz ^1^H, 126 MHz ^13^C) spectrometer. The solvents were CDCl_3_ (TMS as internal standard) and methanol-d_4_. High-resolution mass spectrometry (HRMS) was measured on Agilent 6230 mass spectrometer (ESI as ion source). The fluorescent spectrum was obtained using Shimadzu RF6000 fluorescence spectrophotometer. The absorbance was measured using Bio-Tek Synergy H1 multi-mode microplate reader. Fluorescent images were captured on Zeiss LSM700 laser confocal fluorescence microscope. hNQO1 was purchased from Sigma and NADH was purchased from Aladdin. RPMI 1640 medium, penicillin, streptomycin, and trypsin–EDTA were purchased from Gibco. All chemicals and solvents used in synthesis were purchased commercially and used without further purification unless noted otherwise.

### The Synthesis of Probe 1 and 2

The synthesis of the raw material trimethyl-locked quinone propionic acid (Q_3_PA) refers to the synthesis methods in the existing literature. The synthesis of probe **1** and **2** can be available in Supporting Information.

### Sensitivity of Probe 1 and 2 to NQO1

The probe is dissolved in DMSO to prepare a probe stock solution with a concentration of 10 mM for use, and stored in a refrigerator at 4°C. The NADH (100 μM) and the hNQO1 solutions (0.0625, 0.125, 0.25, 0.5, 1, 2, 4, 8, and 16 μg/mL) were prepared, respectively. The probe stock solution was diluted to 10 μM with PBS (10 mM, pH = 7.4) containing 0.025% DMSO. Then 100 μM NADH and different concentrations of NQO1 (0–16 μg/mL) are added to mix evenly and placed in a water bath at 37°C for 10 min. For all fluorescence spectroscopy studies, the fluorescent emission spectrum from 450 to 600 nm were measured and recorded with an excitation wavelength of 435 nm.

### Selectivity of Probe 1 and 2 to NQO1

Another important parameter for evaluating the performance of newly designed fluorescent probes is high selectivity. Therefore, in order to evaluate the selectivity of these two probes, ions (Ca^2+^, K^+^, Mg^2+^, Na^+^, Cl^−^), amino acids (Arg, Ala, Cys, Gly, GSH, His, Tyr) and NADH were selected as detection targets. The probe stock solution was diluted to 10 μM in PBS (10 mM, pH = 7.4) containing 0.025% DMSO, and various interference reagents (100 μM) prepared in advance were added. The mixed solution was placed in a water bath at 37°C and reacted for 10 min. The fluorescent emission spectrum from 450 to 600 nm were measured and recorded with an excitation wavelength of 435 nm.

### Cytotoxicity Assay

A549 cell culture: 10% fetal bovine serum (FBS) and 1% penicillin-streptomycin were added to the 1640 medium and mixed as the culture medium for A549 cells. A549 cells with appropriate density and culture medium were added to T-25 flasks and cultured in a constant temperature incubator (37°C, 5% CO_2_). During passage, cells were digested with 0.25% trypsin digestion solution (containing 0.02% EDTA). The supernatant was discarded by centrifugation, and then medium was added to resuspend the cells. Finally, a small amount of cells was added to the T-25 flask for cultivation.

The CCK-8 experiment was used to evaluate the cytotoxicity of these two probes. The grown A549 cells were seeded into 96-well plates (10,000 cells/well) and incubated in a constant temperature incubator for 24 h. After discarding the original medium, 100 μL of 1640 medium containing different concentrations of probe (0, 2, 4, 6, 8, 12, 16, and 20 μM) was added and incubated for 24 h. The medium was then discarded and rinsed three times with PBS solution, and each well was filled with 1640 medium containing CCK-8 solution. After incubating the 96-well plate for about 20–60 min, the absorbance at 490 nm was measured. The cell viability was calculated using the following formula.

$$\begin{array}{l} \text{Cellviability}\left(\text{\%}\right)=\left(\text{As}-\text{Ab}\right)/\left(\text{Ac}-\text{Ab}\right)\times 100\% \end{array}$$

As: absorbance of cells incubated with probes of various concentrations; Ac: absorbance of cells incubated with 0 μM probe; Ab: absorbance of wells with medium containing CCK-8 solution.

### Bioimaging Application

The grown A549 cells were seeded into a confocal dish at an appropriate density and divided into two groups: the probe group and the dicoumarin group. Both groups of cells were cultured in a constant temperature incubator for 24 h. The medium in the dicoumarin group was discarded, and then the medium containing 100 μM dicoumarin (DIC) was added and incubated for 12 h. After discarding the original medium of these two groups of cells, the medium containing 20 μM probe was added to continue incubation for 3 h. After rinsing with PBS solution for three times, fluorescent images were taken under a confocal laser scanning microscopy and fluorescent signals were recorded.

## Results and Discussion

### Synthesis Route of the Probe 1 and Probe 2

**Scheme 1:**
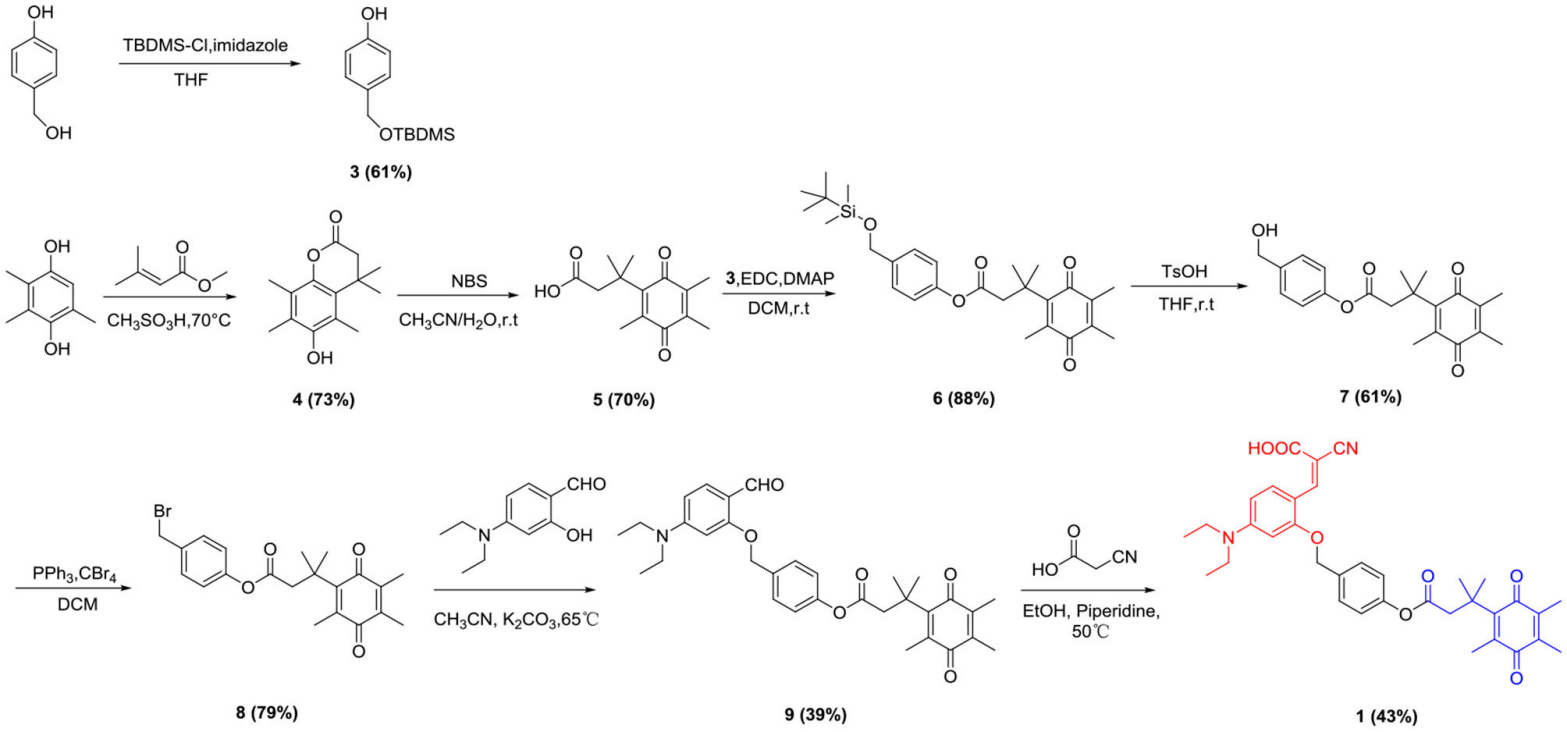
Synthetic route of probe **1**.

**Scheme 2:**
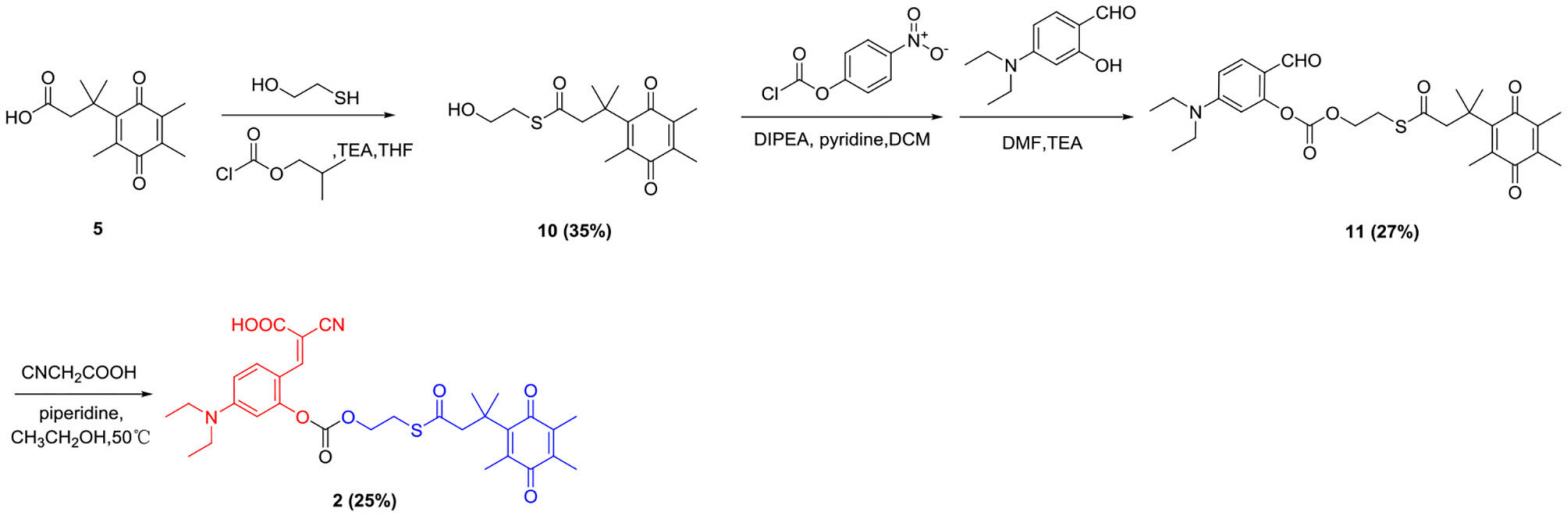
Synthetic route of probe **2**.

Trimethyl-locked quinone propionic acid (Q_3_PA) is an excellent enzyme substrate group, which can be quickly recognized and reduced by the NQO1 enzyme and undergo an intramolecular esterification reaction, which can quickly release the fluorescent group. Therefore, Q_3_PA was selected as the recognition group of the probe **1** and **2**. Covalent-assembly based probes are in a non-fluorescent state before being triggered. After being activated by specific enzymes, fluorescent reporter groups are formed *in situ*. Such probes have high target background ratio and sensitivity and coumarin which was commonly used as organic fluorescent group has excellent spectral properties. Therefore, the coumarin precursor compound 2-cyano-3-(4-(diethylamino)-2-hydroxyphenyl) acrylic acid was selected as fluorescent signal group and was connected to Q_3_PA via p-hydroxybenzyl alcohol or 2-mercaptoethanol linker to design and synthesize novel fluorescent probe **1** and probe **2** for detecting hNQO1. The detailed synthesis of probe **1** was outlined in Scheme 1 and the detailed synthesis of probe **2** was outlined in Scheme 2. The structures of all intermediates and the final product were confirmed by nuclear magnetic resonance (NMR) and high resolution mass spectrometry (HRMS).

### Spectrum Properties

It has been reported that NADH can react with dyes such as rhodamine, methylene blue, and resorcinol, thereby interfering with the fluorescent detection of NQO1 (Best et al., 2016). However, as shown in Figure S1, probe **1** and probe **2** showed only weak fluorescence with a maximum excitation wavelength at 435 nm, and the mixture with NADH also showed slightly enhanced fluorescence which indicated that the determination of hNQO1 by utilizing probe **1** and **2** will not be interfered by NADH. And absorbance spectra of probe 1 (A) and probe 2 (B) in the presence of hNQO1 and NADH were also investigated (Figure S2).

**Figure 1:**
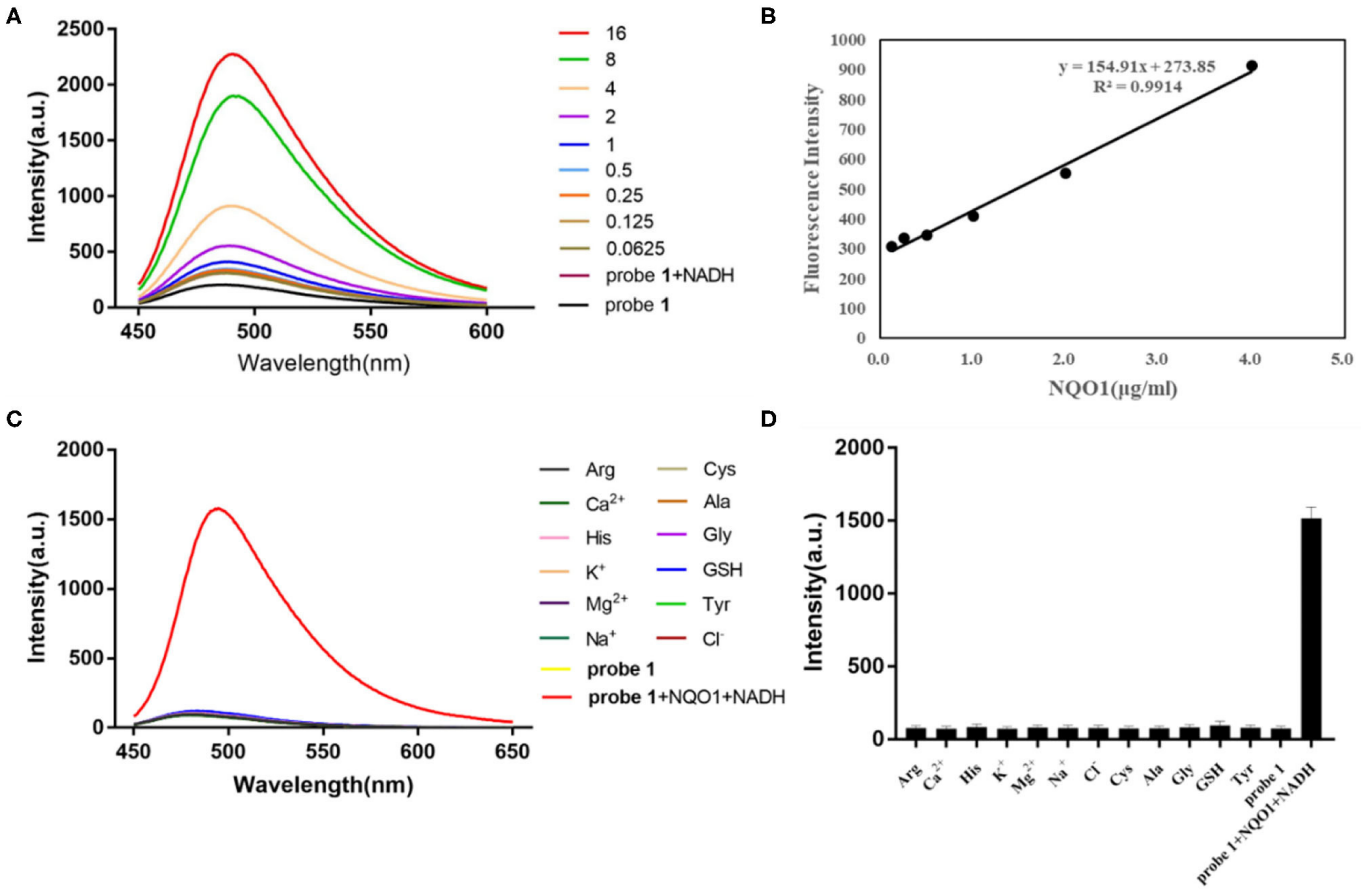
**(A)** Fluorescent spectrum of probe **1** (10 μM) with different concentrations of hNQO1 (0–16 μg/mL) and NADH (100 μM) in PBS buffer (10 mM, pH = 7.4). λ_ex_ = 435 nm, excitation slit width = 5.0 nm, emission slit width = 3.0 nm. **(B)** Linear relationship (*R*^2^ = 0.9914) between the fluorescent intensity of probe **1** at 490 nm and the concentration of hNQO1 (0.125–4 μg/mL). **(C)** Fluorescence responses of probe **1** (10 μM) upon adding with NQO1 (16 μg/mL), NADH (100 μM) and other biologically-relevant species (100 μM) in PBS buffer (10 mM, pH = 7.4). **(D)** Bars represent the fluorescent intensity of probe **1** at 490 nm with hNQO1 and other biologically relevant substances.

To further explore the ability of probe **1** and probe **2** for detecting hNQO1, their sensitivity to hNQO1 under the optimal enzyme reaction conditions were investigated. Probe **1** (10 μM) and probe **2** (10 μM) were mixed with NADH (100 μM) and different concentrations of hNQO1 (0–16 μg/mL), respectively. The fluorescent spectrum was measured. As shown in Figure 1A, the fluorescent intensity of probe **1** was extremely weak under excitation at 435 nm. After adding 0.5 μg/mL hNQO1, the fluorescence at 490 nm began to change significantly and increased with the addition of hNQO1. When the concentration of hNQO1 was 16 μg/mL, the fluorescent intensity increased about 10-folds. In addition, the fluorescence intensity of probe **1** at 490 nm has a good linear relationship (*R*^2^ = 0.9914) with the concentration of hNQO1 (0.125–4 μg/mL) (Figure 1B).

**Figure 2:**
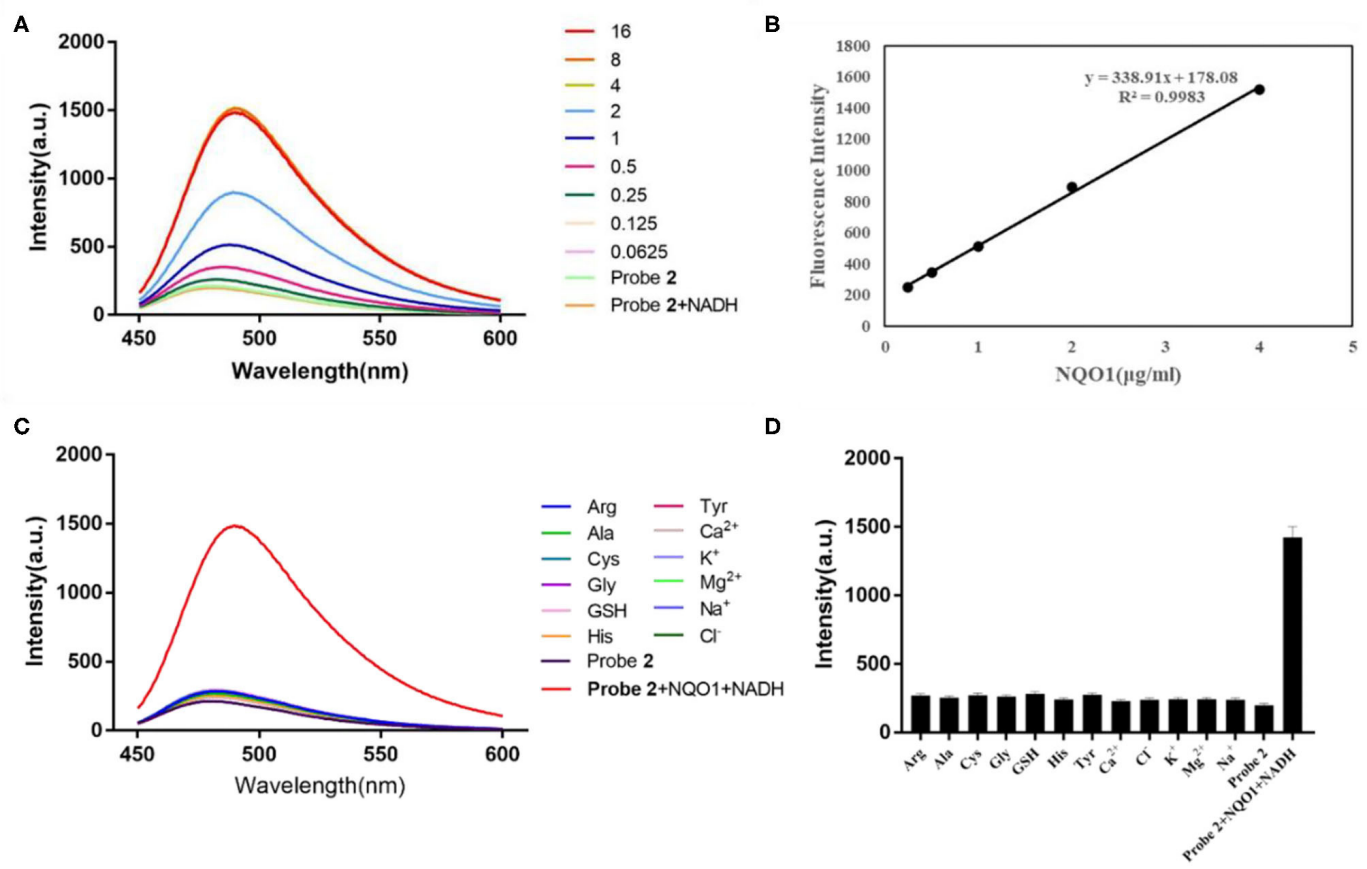
**(A)** Fluorescent spectrum of probe **2** (10 μM) after adding different concentrations of hNQO1 (0–16 μg/mL) and NADH (100 μM) in PBS buffer (10 mM, pH = 7.4). λ_ex_ = 435 nm, excitation slit width = 3.0 nm, emission slit width = 5.0 nm. **(B)** Linear relationship (*R*^2^ = 0.9983) between the fluorescent intensity of probe **2** at 490 nm and the concentration of hNQO1 (0.125–4 μg/mL). **(C)** Fluorescence responses of probe **2** (10 μM) upon adding with NQO1 (16 μg/mL), NADH (100 μM) and other biologically-relevant species (100 μM) in PBS buffer (10 mM, pH = 7.4). **(D)** Bars represent the fluorescent intensity of probe **2** at 490 nm with hNQO1 and other biologically relevant substances.

Similarly, the fluorescent intensity of probe **2** at 490 nm increased sharply with the addition of hNQO1 (Figure 2). When the concentration of hNQO1 was 8 μg/mL, the fluorescent intensity was close to the peak and increased by nearly 7-folds compared with the fluorescence generated by probe itself. Moreover, a linear functional relationship (*R*^2^ = 0.9983) was obtained between the concentration of hNQO1 (0.25–4 μg/mL) and the fluorescence intensity of probe **2** at 490 nm. These results indicated that probe **1** and probe **2** have high sensitivity to NQO1, and can be used for further selective experiments and biological imaging applications.

### Selectivity Evaluation

Another important parameter for evaluating the performance of newly designed fluorescent probes is high selectivity. It can be clearly observed in Figure 1C that the fluorescence spectrum of the solution did not show significant changes when probe **1** was incubated with interfering substances other than hNQO1. However, probe **1** incubated with hNQO1 showed a significant fluorescence emission peak at 490 nm, and the fluorescence intensity was significantly enhanced. In addition, Figure 1D showed that the change in fluorescence intensity of probe **1** caused by hNQO1 was nearly 20 times that of other interferences. Similarly, except for hNQO1, the fluorescence of probe **2** after adding other interfering substances was almost unchanged, and the fluorescent intensity of probe **2** caused by hNQO1 is nearly 6 times that of other interferents. These results indicated that regardless of the presence of other biologically relevant substances, probe **1** and probe **2** can be used as fluorescent turn-on probes to detect the activity of hNQO1 in cells with high selectivity.

### Reaction Mechanism

**Scheme 3:**
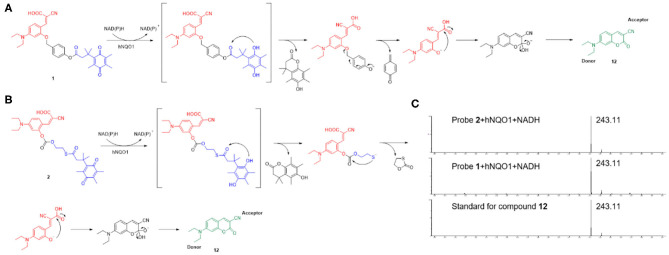
The potential response mechanism of probe **1 (A)** and probe **2 (B)** for hNQO1 detection and the structure of compound **12**. **(C)** HRMS spectra of standard for compound **12** and the reaction mixture of probe **1** and probe **2** with the addition of hNQO1 and NADH.

As shown in Scheme 3, probe **1** and probe **2** had almost no fluorescence. In the presence of NADH, hNQO1 played a role of two-electron reduction, reducing the quinone in probe **1** to hydroquinone, and then a cascade reaction occurred. The self- cleavage of the connecting linker led to the release of 4-methylenecyclohex-2,5-dien-1-one and the *in-situ* formation of the fluorescent compound 7-diethylamino-3-cyanocoumarin (compound **12**). Compound **12** is composed of three parts, an electron donor part composed of a diethylamino group, a conjugated part composed of benzopyran, and an electron acceptor part composed of a carbonyl group and a cyano group. This D-π-A structure makes it have excellent spectral properties. The mechanism of probe **2** for selective identification and detection of hNQO1 is similar to that of probe **1**. After responding to hNQO1, 1,3-oxathiolan-2-one and 7-diethylamino-3-cyanocoumarin were released and the fluorescence turned on. In addition, the results of HRMS confirmed the mechanism of probe **1** and probe **2** with the addition of hNQO1 and NADH. Fluorescent product compound **12** can be detected in the solution after the reaction of these two probes with hNQO1.

### Cytotoxicity Evaluation

**Figure 3:**
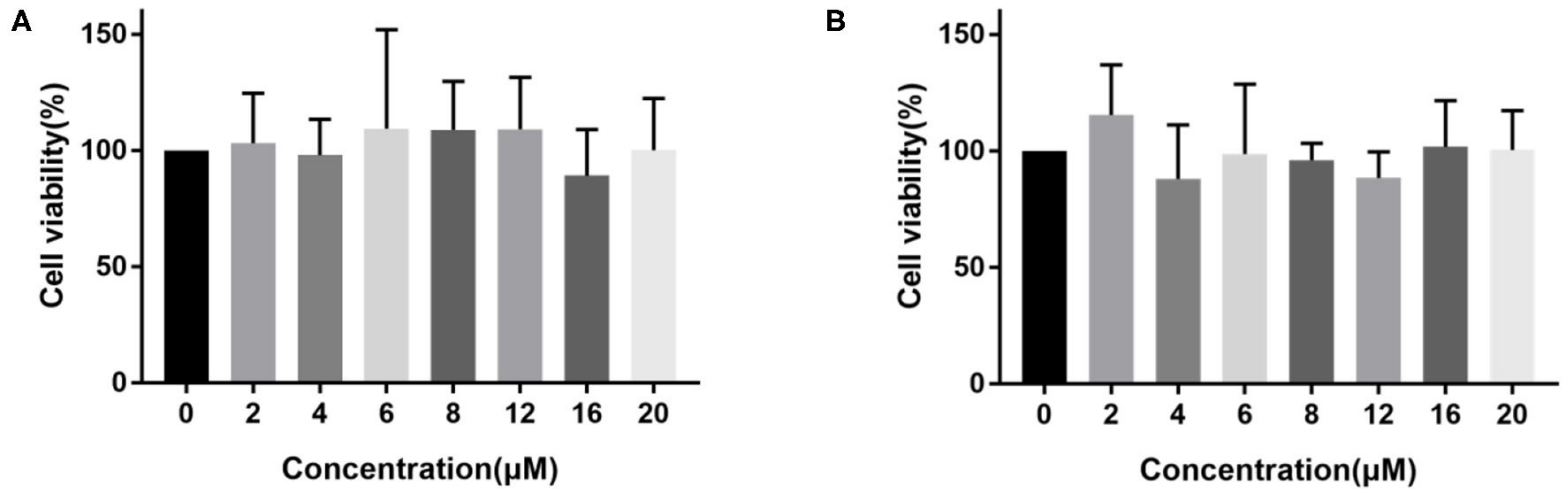
The viability of A549 cells incubated with different concentrations (0–20 μM) of probe **1 (A)** and probe **2 (B)**.

Considering the effectiveness and selectivity of probe **1** and probe **2** in detecting the activity of hNQO1 *in vitro*, we further studied the ability of these two probes to identify and distinguish tumor cells from normal cells with imaging in living cells. First, in order to evaluate the compatibility of the probes with biological systems, we used the CCK-8 method to investigate the toxic effects of these two probes on A549 cells. As shown in Figure 3, the viability of A549 cells incubated with probe **1** or probe **2** at different concentrations was above 80%. These results indicated that probe **1** and probe **2** have low toxicity and can be further used for biological imaging applications.

### Fluorescence Imaging in Cancer Cells

We next investigate the ability of these two probes to detect hNQO1 in living cells. A549 cells with high expression of hNQO1 were selected as detection objects and co-incubated with the probe for fluorescence confocal imaging. At the same time, A549 cells were pretreated with the hNQO1 inhibitor dicoumarol and co-incubated with the probe for fluorescence confocal imaging.

**Figure 4:**
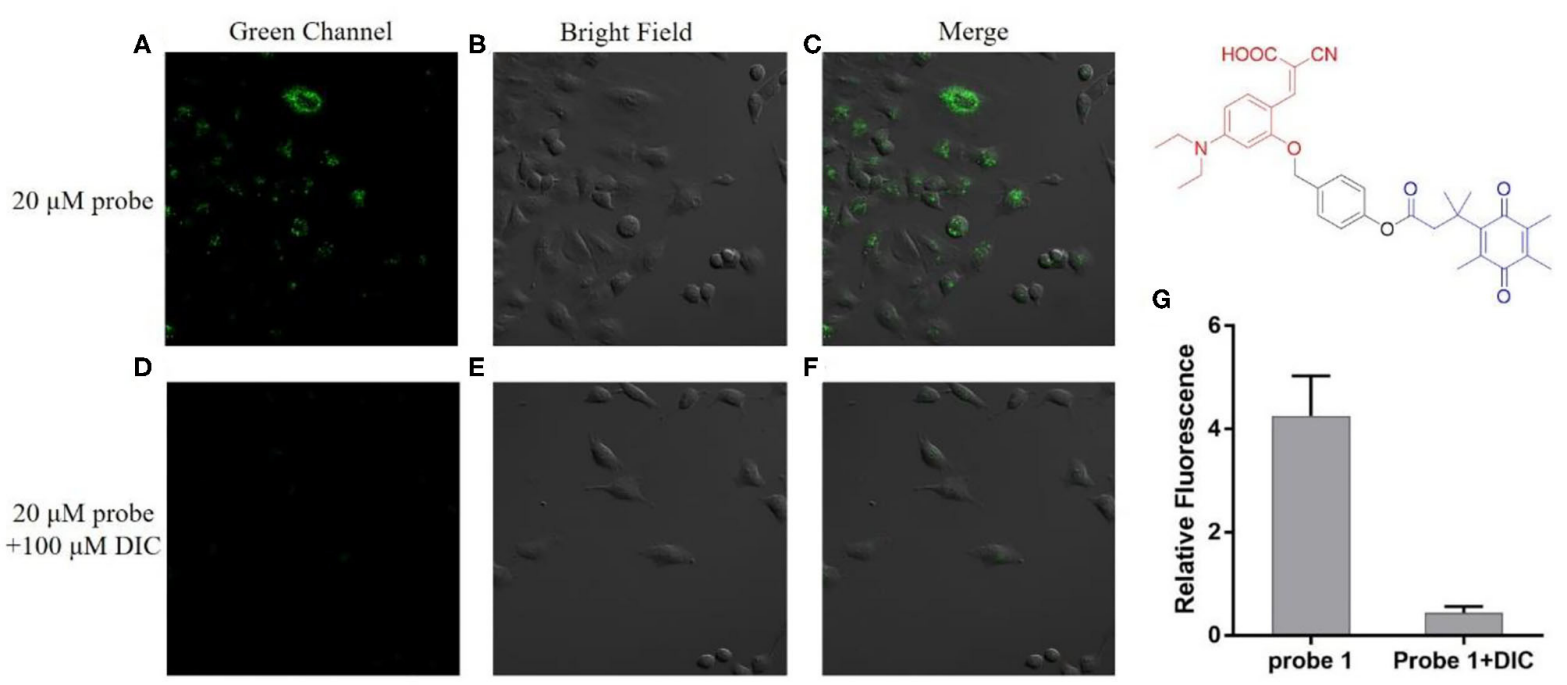
Fluorescence confocal imaging of A549 cells after incubation with probe **1 (A–C)** and A549 cells pretreated with dicoumarol after incubation with probe **1 (D–F)**. λex = 488 nm. **(G)** Relative fluorescence intensity of the images from **(A–F)**.

**Figure 5:**
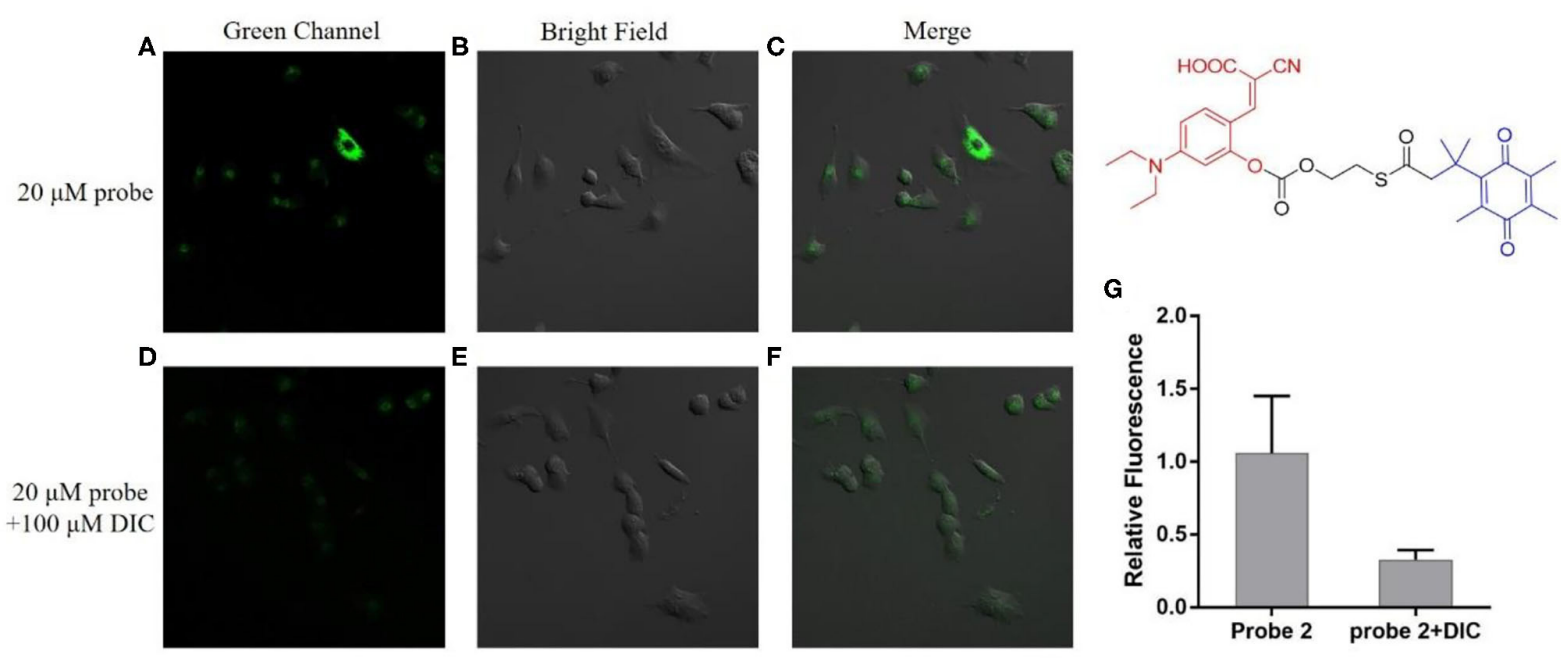
Fluorescence confocal imaging of A549 cells after incubation with probe **2 (A–C)** and A549 cells pretreated with dicoumarol after incubation with probe **2 (D–F)**. λex = 488 nm. **(G)** Relative fluorescence intensity of the images from **(A–F)**.

As can be seen from Figure 4, the A549 cells incubated with probe **1** showed obvious green fluorescence, and the fluorescence was mainly distributed in the cytoplasm. This indicates that probe **1** can penetrate the cell membrane into the cell and can also be triggered by hNQO1 to form a coumarin derivative *in situ*, releasing strong fluorescence. In contrast, after the cells were treated with dicoumarol, the hNQO1 activity in the cells was significantly inhibited. The lack of hNQO1 activation caused probe **1** to be unable to form a fluorophore *in situ* and eventually showed only weak fluorescence. This also confirmed that probe **1** can only be activated in the presence of hNQO1, resulting in an enhanced fluorescence signal. Similarly, as can be seen from Figure 5, probe **2** also showed significant green fluorescence in A549 cells, while the control group showed only weak fluorescence. In conclusion, probe **1** and probe **2** have good selectivity and sensitivity to hNQO1 in living cells, and are expected to be used for early diagnosis of cancer.

## Conclusion

In summary, based on the principle of covalent assembly, we used NQO1-specific Q_3_PA as the recognition group, coumarin precursor (Z)-2-cyano-3-(4-(diethylamino)-2-hydroxyphenyl)acrylic acid as the fluorescent signal group, and p-hydroxybenzyl alcohol or 2-mercaptoethanol as the connecting linker to design and synthesize the enzyme-activated fluorescent probe **1** and probe **2**. These two probes were excellent in selectivity and sensitivity. They could be quickly recognized and reduced by NQO1, and were not interfered by NADH and other biological related substances including ions and amino acids. Probe **1** and probe **2** showed weak fluorescence when excited at 435 nm. When they were incubated with NQO1 and NADH, an obvious emission peak at 490 nm was observed at the same excitation wavelength, and the fluorescent intensity increased by nearly 7–10 times. This change was due to the *in-situ* formation and release of the fluorescent compound 7-diethylamino-3-cyanocoumarin. Probe **1** has a good linear relationship with the concentration of hNQO1 in the range of 0.125–4 ug/ml, while probe **2** has a linear relationship with the concentration of hNQO1 in the range of 0.25–4 ug/ml. These two probes have good water solubility, good stability, and low toxicity to cells. In addition to quantitative detection of hNQO1 activity *in vitro*, they have also been used in fluorescence imaging in living cells successfully. Compared with dicoumarol-treated A549 cells, probes **1** and **2** showed significantly enhanced green fluorescent signals in A549 cells which have high expression of NQO1. This result shows that the human cancer cells with different hNQO1 activity levels can be easily detected and differentiated according to the fluorescent intensity displayed by probe **1** and probe **2**. Therefore, NQO1-activated probe **1** and probe **2** have better application prospects in early clinical diagnosis and evaluation of drug efficacy in preclinical studies.

## Data Availability Statement

The raw data supporting the conclusions of this article will be made available by the authors, without undue reservation.

## Author Contributions

XX and CG directed the experiments and wrote the paper. JH and LC did the experiments. YZ helped JH in some experiments. All authors contributed to the article and approved the submitted version.

## Conflict of Interest

The authors declare that the research was conducted in the absence of any commercial or financial relationships that could be construed as a potential conflict of interest.
