# Supplementary material for: Covalent-Assembly Based Fluorescent Probes for Detection of hNQO1 and Imaging in Living Cells
Source: Front Chem. 2020 Aug 26;8:756. doi: 10.3389/fchem.2020.00756 (PMC7479225; doi:10.3389/fchem.2020.00756)
Supplement: Supplementary file 1 [file Presentation_1.pdf]

Supporting Information for

Covalent-assembly based fluorescent probes for detection of hNQO1 and imaging in living cells

Jialing Han [b,1], Longhao Cheng [a,1], Ya Zhu [c], Xiaowei Xu [c,*], Chaoliang Ge [a,*]

[a] Departmentof Pharmacy, the First Affiliated Hospital of Anhui Medical University

[b] Hai Men People's Hospital, 226100, Nantong, China.

[c] School of Pharmacy, China Pharmaceutical University, 210009, Nanjing, China.

[1] These authors contribute equally to this work.

[*] Corresponding Authors: xw@cpu.edu.cn (Xiaowei Xu); gechaoliang@126.com (Chaoliang Ge).

1. Synthesis of Probe 1

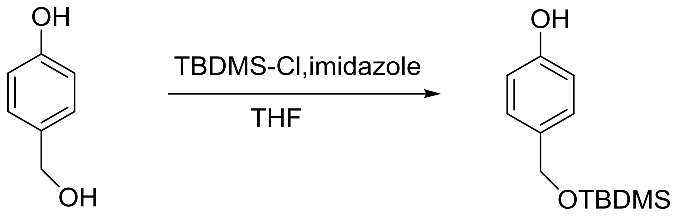

3 (61%)

Synthesis of compound 3. The raw material p-hydroxybenzyl alcohol (1 g, 8.06 mmol) was dissolved in 5 mL of tetrahydrofuran, imidazole (658 mg, 9.66 mmol) was added, and the reaction was carried out at 0 ℃ for 30 min. Then keeping the temperature unchanged, tert-butyldimethylchlorosilane (TBDMS-Cl, 1.5 g, 9.95 mmol) was added. And then the solution was moved to room temperature and stirred for 2 h. After the reaction was completed, ether was added and washed with saturated ammonium chloride water three times, then dried over anhydrous Na_2SO_4. The crude product (1.8 g) was separated by column chromatography (PE: EA = 24:1) to obtain a transparent liquid (1.17 g, 61%). ^{1}H NMR (300 MHz, Chloroform-d) δ 9.78 (s, 1H), 8.11 (d, J = 8.0 Hz, 2H), 7.45 (d, J = 8.0 Hz, 2H), 4.84 (s, 2H), 0.98 (s, 9H), 0.14 (s, 6H).

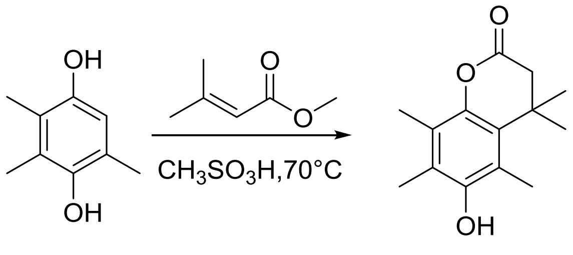

4 (73%)

Synthesis of compound 4. The raw material trimethylhydroquinone (4 g, 26.28 mmol) was dissolved in methanesulfonic acid (32 mL). Methyl 3,3-dimethacrylate (2.2 mL, 16.83 mmol) was added and the reaction was stirred at 70 ℃ for 2.5 h. After the reaction was completed, it was cooled to room temperature and slowly poured into water at 0 ℃. Then appropriate amount of ethyl acetate was added. After standing for separation, the organic phase was washed three times with water, one times with saturated $NaHCO_3$ and saturated NaCl, and dried over anhydrous Na_2SO_4. Then the crude product (7 g)

was separated by silica gel column chromatography (PE: DCM = 2:1) and recrystallized from petroleum ether. After purification, pure white solid (4.5 g, 73%) was obtained. ^{1}H NMR (500 MHz, chloroform-d) δ 4.62 (s, 1H), 2.60 (s, 2H), 2.40 (s, 3H), 2.27 (s, 3H), 2.23 (s, 3H), 1.50 (s, 6H).

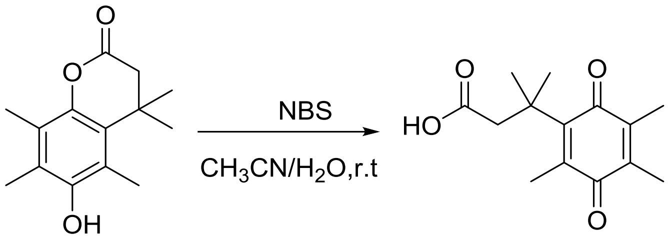

4 **5 (70%)**

Synthesis of compound 5. Compound **4** (3.6 g, 15.36 mmol) was dissolved in 181 mL of acetonitrile. Then 77 mL of water and N-bromosuccinimide (NBS, 3 g, 16.86 mmol) were added and stirred at room temperature for 1 h. The reaction solution was distilled off a part of the organic solvent by a rotary evaporator. The remaining reaction solution was extracted three times with ethyl acetate, washed with saturated NaCl, and dried over anhydrous Na_2SO_4. The crude product (5 g) was separated by silica gel column chromatography (PE: EA = 12:1) to obtain a yellow solid (2.7 g, 70%).^{1}H NMR (300 MHz, chloroform-d) δ 3.04 (s, 2H), 2.16 (s, 3H), 1.97 (s, 3H), 1.95 (s, 3H), 1.46 (s, 6H).

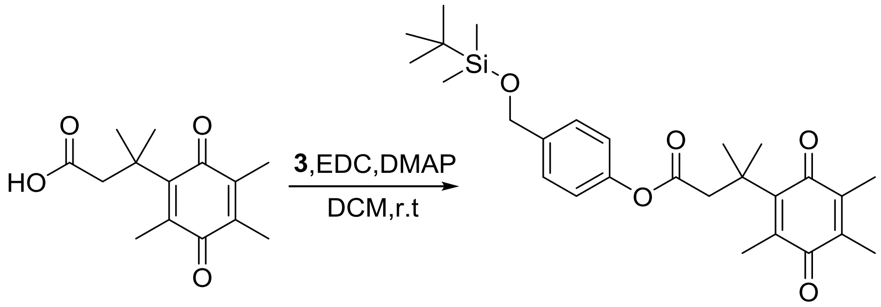

5 **6 (88%)**

Synthesis of compound 6. Compound **5** (1.5 g, 4.91 mmol), Compound **3** (1.17 g, 4.91 mmol), 1-(3-dimethylaminopropyl)-3-ethylcarbodiimide hydrochloride (EDCI, 1.9 g, 9.91 mmol) and 4-dimethylaminopyridine (DMAP, 1.2 g, 9.82 mmol) were added to a

25 mL eggplant bottle in sequence. 5 mL of re-distilled anhydrous dichloromethane was dissolved and reacted overnight at room temperature under the protection of nitrogen. The reaction solution was extracted with DCM, washed with saturated NaCl, and dried over anhydrous Na_2SO_4. The crude oil (3.5 g) was separated by silica gel column chromatography (PE: EA = 24: 1) to obtain yellow solid (2.03 g, 88%). ^{1}H NMR (300 MHz, Chloroform-d) δ 7.30 (d, J = 9.3 Hz, 2H), 6.95 (d, J = 8.3 Hz, 2H), 4.72 (s, 2H), 3.25 (s, 2H), 2.20 (s, 3H), 1.94 (s, 6H), 1.55 (s, 6H), 0.95 (s, 9H), 0.11 (s, 6H).

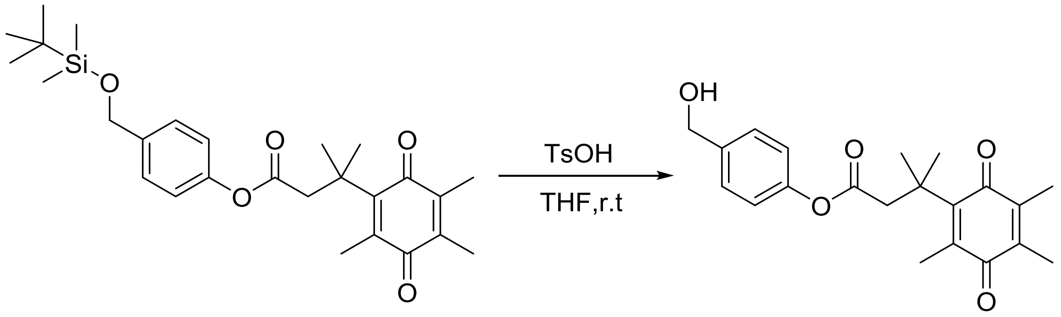

6 7 (61%)

Synthesis of compound 7. Compound **6** (1.96 g, 4.16 mmol) was dissolved in 5 mL of re-distilled anhydrous tetrahydrofuran. P-toluenesulfonic acid (397 mg, 2.09 mmol) was added at 0 ℃, and the reaction solution was moved to room temperature and reacted overnight. The organic solvent in the reaction solution was spin-dried by a rotary evaporator, and the remaining solution was dissolved in DCM and washed three times with water. Subsequently, the organic layer was washed with saturated NaCl and dried over anhydrous s Na_2SO_4. The crude oil (1.86 g) was separated by silica gel column chromatography (PE: EA = 16:1~8:1) to obtain a yellow oil (898 mg, 61%). ^{1}H NMR (400 MHz, Chloroform-d) δ 7.36 (d, J = 8.6 Hz, 2H), 6.99 (d, J = 8.5 Hz, 2H), 4.68 (s, 2H), 3.26 (s, 2H), 2.19 (s, 3H), 1.94 (d, J = 3.9 Hz, 6H), 1.55 (s, 6H).

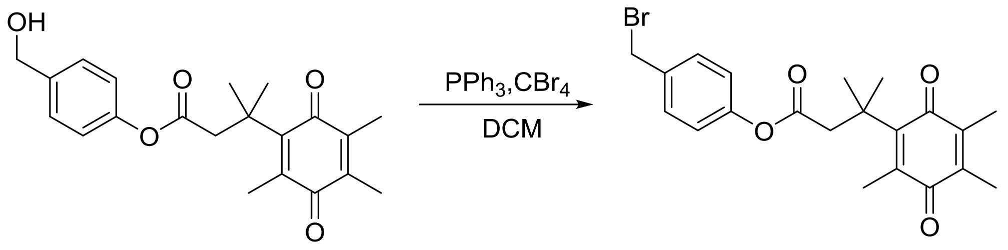

7 **8 (79%)**

Synthesis of compound 8. Compound **7** (655 mg, 1.84 mmol) and carbon tetrabromide (CBr_4, 732 mg, 2.21 mmol) were dissolved in 2 mL of anhydrous DCM. Triphenylphosphine (PPh_3, 579 mg, 2.21 mmol) in DCM (2 mL) was added at 0 ℃. Then the liquid mixture was moved to room temperature and reacted overnight. After completion, the solution was extracted with DCM, washed with saturated NaCl, and dried over anhydrous Na_2SO_4. The crude product (1.6 g) was separated by silica gel column chromatography (PE: EA = 24:1) to obtain compound **8** (609 mg, 79%). ^{1}H NMR (300 MHz, Chloroform-d) δ 7.38 (d, *J* = 8.3 Hz, 2H), 6.97 (d, *J* = 8.3 Hz, 2H), 4.48 (s, 2H), 3.26 (s, 2H), 2.19 (s, 3H), 1.95 (s, 6H), 1.54 (s, 6H).

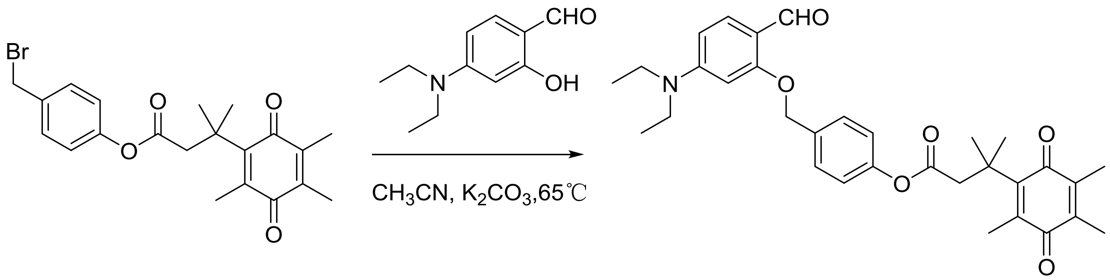

8 **9 (39%)**

Synthesis of compound 9. Compound **8** (723 mg, 1.72 mmol), 4-(diethylamino)-2-hydroxybenzaldehyde (334 mg, 1.72 mmol) and potassium carbonate (715 mg, 5.17 mmol) were dissolved in 69 mL of acetonitrile. The mixed liquid was heated to reflux at 65 ℃ for 12 h under nitrogen protection. After the solvent was spin-dried, the remaining solution was extracted three times with DCM, washed once with saturated NaCl, and dried over anhydrous Na_2SO_4. The obtained crude product was separated by silica gel column chromatography (PE: EA = 24:1~8:1) to obtain compound **9** (355 mg, 39%). ^{1}H NMR (300 MHz, Chloroform-d) δ 10.22 (s, 1H), 7.74 (d, *J* = 8.9 Hz, 1H),

7.44 (d, J = 8.1 Hz, 2H), 7.03 (d, J = 8.3 Hz, 2H), 6.31 (d, J = 8.9 Hz, 1H), 6.04 (s, 1H), 5.14 (s, 2H), 3.40 (q, J = 6.8 Hz, 4H), 3.27 (s, 2H), 2.19 (s, 3H), 1.95 (s, 6H), 1.55 (s, 6H), 1.18 (t, J = 7.0 Hz, 6H).

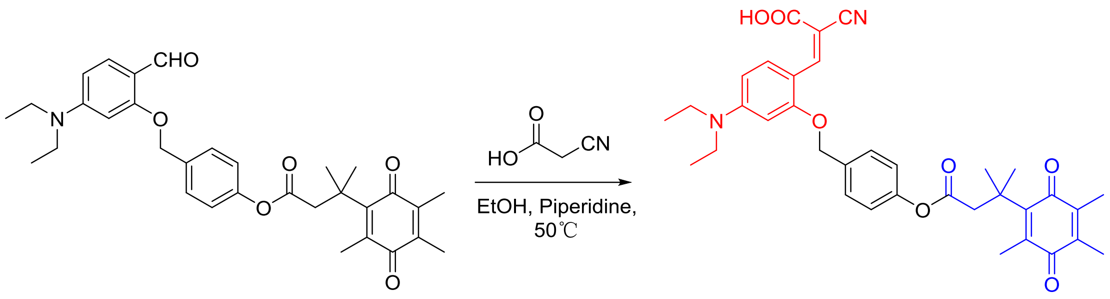

9 **1 (43%)**

Synthesis of probe 1. Compound **9** (170 mg, 0.32 mmol), cyanoacetic acid (54 mg, 0.64 mmol), piperidine (0.12 mL, 1.30 mmol) and acetic acid (0.12 mL, 2.10 mmol) were dissolved in 25 mL of ethanol. The mixed liquid was heated to reflux at 50 ℃ for 12 h under nitrogen protection. After the solvent was spin-dried, the remaining reaction solution was extracted three times with ethyl acetate, washed with saturated NaCl, and dried over anhydrous Na_2SO_4. The obtained crude product was separated by silica gel column chromatography (PE: EA = 1:1) to obtain probe **1** (85 mg, 43%). ^{1}H NMR (300 MHz, Methanol-d4) δ 8.66 (s, 1H), 8.28 (d, J = 9.0 Hz, 1H), 7.49 (d, J = 7.9 Hz, 2H), 7.01 (d, J = 8.0 Hz, 2H), 6.39 (d, J = 8.5 Hz, 1H), 6.18 (s, 1H), 5.21 (s, 2H), 3.43 (q, J = 6.9 Hz, 4H), 3.23 (s, 2H), 2.17 (s, 3H), 1.92 (s, 6H), 1.54 (s, 6H), 1.11 (s, 6H). ^{13}C NMR (126 MHz, $CDCl_3$) δ 190.86, 187.44, 171.25, 169.65, 161.36, 153.85, 151.92, 150.24, 148.73, 142.92, 139.23, 138.63, 134.00, 131.73, 127.98, 121.98, 118.13, 109.46, 105.70, 94.53, 90.73, 69.93, 47.69, 45.05, 38.46, 28.98, 14.38, 12.61, 12.14. HRMS m/z: calculated for $C_{35}H_{38}N_2NaO_7$ 621.2571 $(M + Na)^+$, found 621.2564.

2. Synthesis of Probe 2

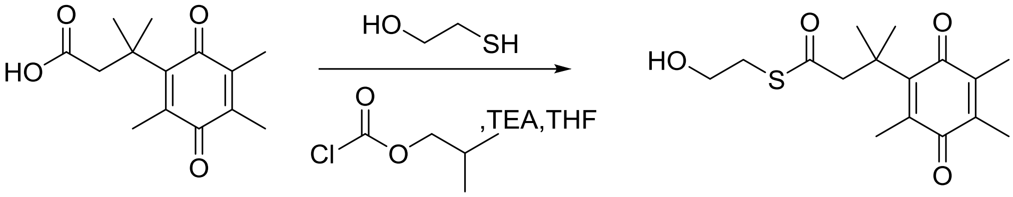

5 **10 (35%)**

Synthesis of compound 10. Compound **5** (1 g, 4.0 mmol) was dissolved in 12 mL of tetrahydrofuran, triethylamine (TEA, 1.2 mL, 8.5 mmol) and isobutyl chloroformate (570 μL, 4.4 mmol) were added at -5 ℃, and stirred for 30 min. 2-Mercaptoethanol (422 μL, 6.0 mmol) and triethylamine (TEA, 0.5 mL, 3.5 mmol) were added immediately. After the addition, the reaction was removed to room temperature for 12 h. After the reaction is completed, suction filtration was carried out, and the filter solid was discarded. After the filtrate was spin-dried, the crude product was obtained and separated by column chromatography (PE: EA = 25:1~16:1) to obtain yellow oil compound **10** (430 mg, 35%). ^{1}H NMR (300 MHz, Methanol-d4) δ 3.55 (t, *J* = 6.6 Hz, 2H), 3.29 (s, 2H), 2.95 (t, *J* = 6.6 Hz, 2H), 2.13 (s, 3H), 1.98 (s, 6H), 1.44 (s, 6H).

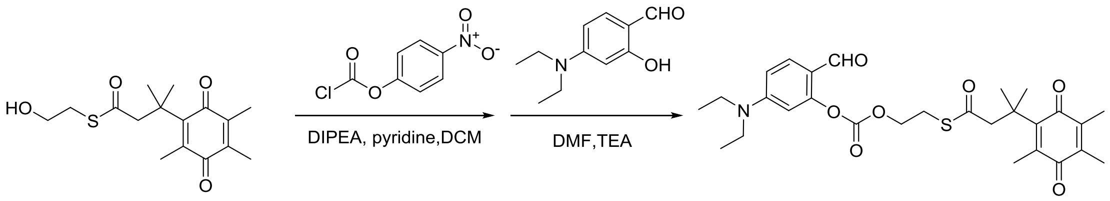

10 **11 (27%)**

Synthesis of compound 11. Compound **10** (354 mg, 1.14 mmol) was dissolved in 13 mL of dichloromethane. 4-Nitrophenylchloroformate (690 mg, 3.42 mmol), N, N-diisopropylethylamine (DIPEA, 0.76 mL, 4.56 mmol) and pyridine (9.2 μL, 0.11 mmol) were added at 0 ℃. The reaction was stirred at room temperature for 5 h. After the reaction, the organic solvent was removed by distillation under reduced pressure. The remaining reaction solution was dissolved with 11 mL of DMF, followed by addition of 4-(diethylamino)-2-hydroxybenzaldehyde (662 mg, 3.42 mmol) and triethylamine (TEA, 1.3 mL, 9.38 mmol), and reacted at room temperature for 24 h. The reaction solution was filtered and the solid was discarded. The filtrate was extracted with ethyl acetate, washed with saturated NaCl, and dried over anhydrous Na_2SO_4. The obtained

crude product was isolated by column chromatography (PE: EA = 16:1~12:1) to obtain compound **11** (201 mg, 27%). ^{1}H NMR (300 MHz, Chloroform-d) δ 9.78 (s, 1H), 7.66 (d, *J* = 8.9 Hz, 1H), 6.59 (dd, *J* = 8.9, 2.4 Hz, 1H), 6.40 (d, *J* = 2.4 Hz, 1H), 4.32 (t, *J* = 6.5 Hz, 2H), 3.44 (q, *J* = 7.1 Hz, 4H), 3.33 (s, 2H), 3.21 (t, *J* = 6.5 Hz, 2H), 2.15 (s, 3H), 1.98 (s, 6H), 1.45 (s, 6H), 1.23 (t, *J* = 7.1 Hz, 6H).

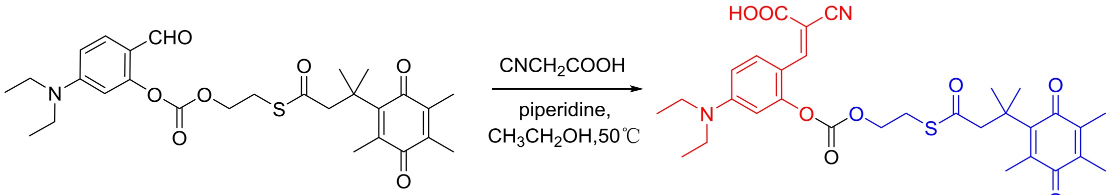

11 **2 (25%)**

Synthesis of probe 2. Compound **11** (69 mg, 0.13 mmol), cyanoacetic acid (22 mg, 0.26 mmol) and piperidine (23 μL, 0.26 mmol) were dissolved in 4 mL of ethanol and heated to reflux under nitrogen at 50 ℃ for 24 h. The organic solvent was spin-dried, and the obtained crude product was separated by silica gel column chromatography (petroleum ether: ethyl acetate = 1:1) to obtain probe **2** (20 mg, 25%). ^{1}H NMR (300 MHz, Methanol-d4) δ 8.39 (d, *J* = 9.2 Hz, 1H), 7.52 – 7.42 (m, 1H), 6.75 (dd, *J* = 9.1, 2.5 Hz, 1H), 6.69 (d, *J* = 2.6 Hz, 1H), 4.36 - 4.28 (m, 2H), 3.50 (q, *J* = 7.1 Hz, 4H), 3.36 (s, 2H), 3.21 (t, *J* = 5.9 Hz, 2H), 2.11 (s, 3H), 1.91 (s, 3H), 1.82 (s, 3H), 1.46 (s, 6H), 1.23 (t, *J* = 7.1 Hz, 6H). ^{13}C NMR (126 MHz, MeOD) δ 198.14, 190.33, 187.07, 153.27, 152.35, 151.74, 147.73, 142.76, 138.85, 129.45, 124.51, 123.80, 118.66, 109.36, 104.29, 67.26, 56.32, 44.45, 38.90, 30.42, 29.39, 28.06, 13.11, 11.46, 10.64. HRMS m/z: calculated for $C_{31}H_{36}N_2NaO_8S$ 619.2085 $(M + Na)^+$, found 619.2074.

3. Spectrophotometric experiments

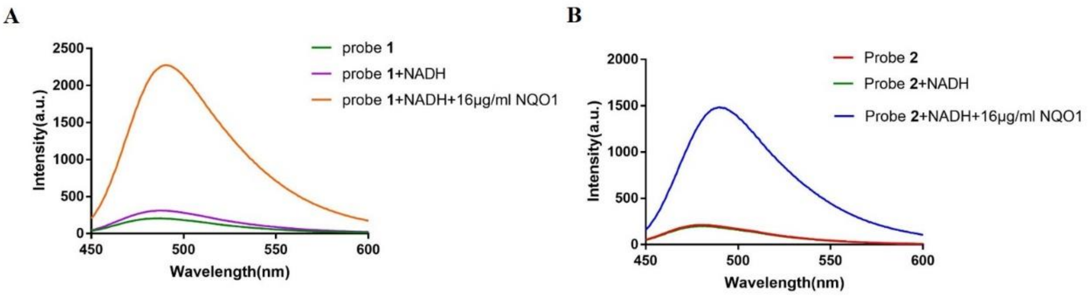

Fig S1. Fluorescence spectra of probe **1** (A) and probe **2** (B) in the presence or absence of NADH and hNQO1.

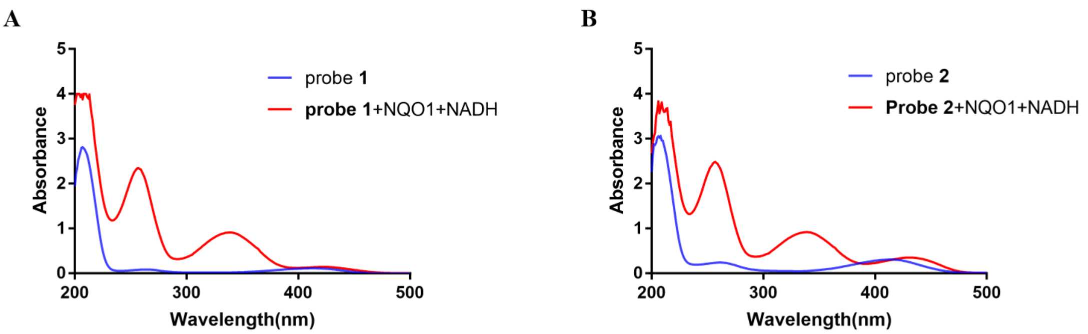

Fig S2. Absorbance spectra of probe **1** (A) and probe **2** (B) in the presence of hNQO1 and NADH.

4. NMR and HRMS Characterizations

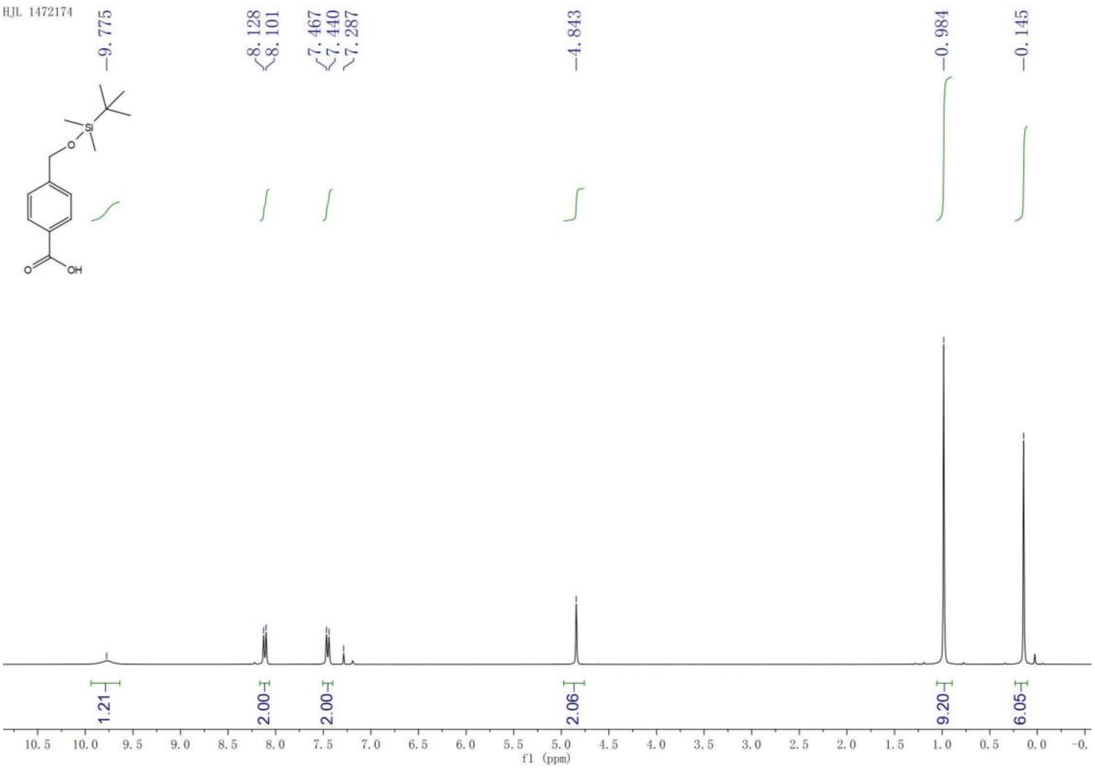

Fig S3. ^{1}H NMR spectrum for compound **3**.

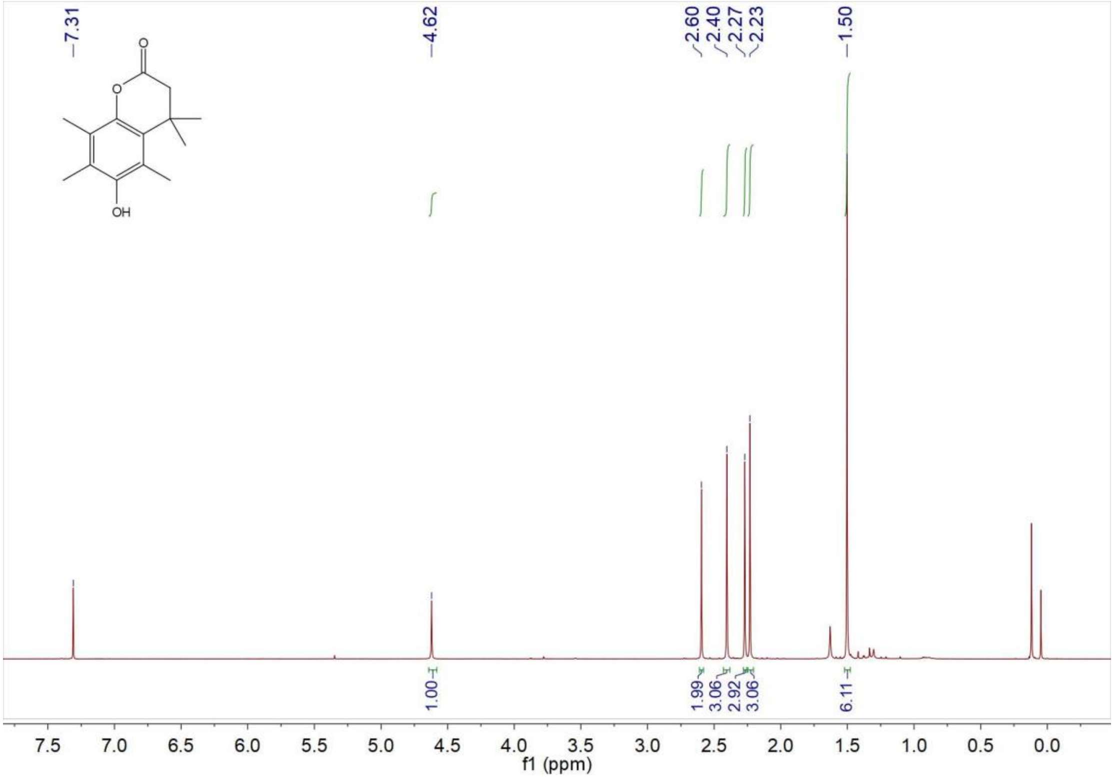

Fig S4. ^{1}H NMR spectrum for compound **4**.

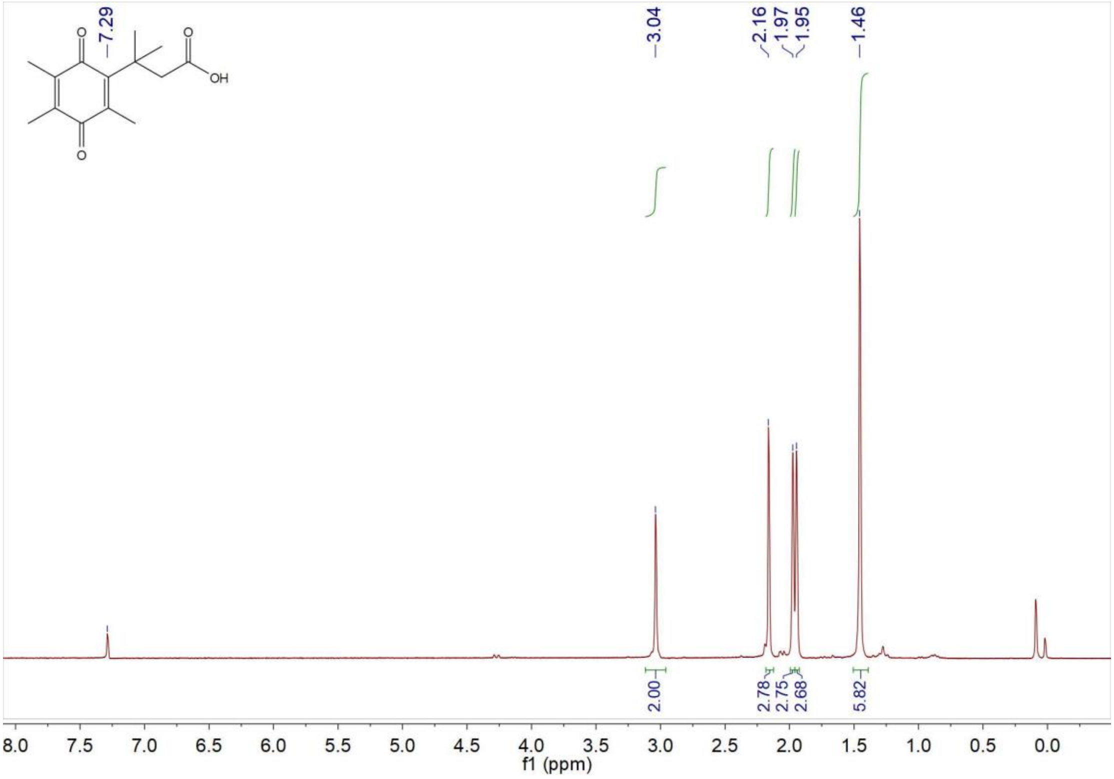

Fig S5. ^{1}H NMR spectrum for compound **5**.

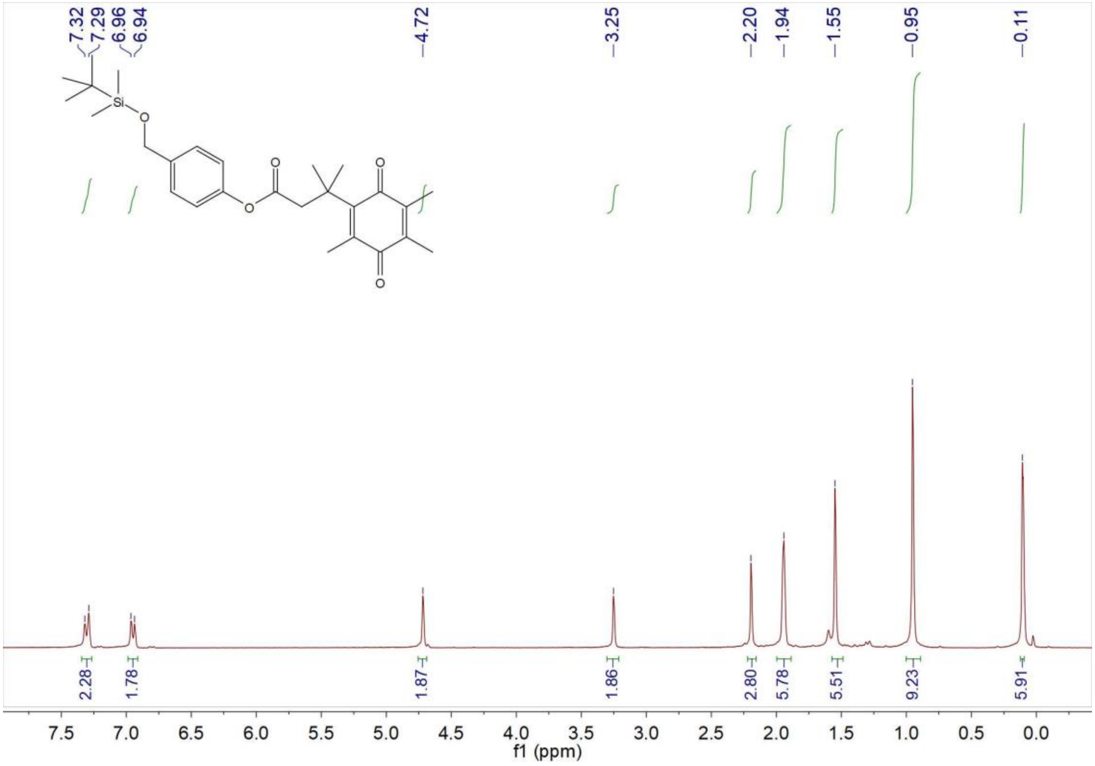

Fig S6. ^{1}H NMR spectrum for compound **6**.

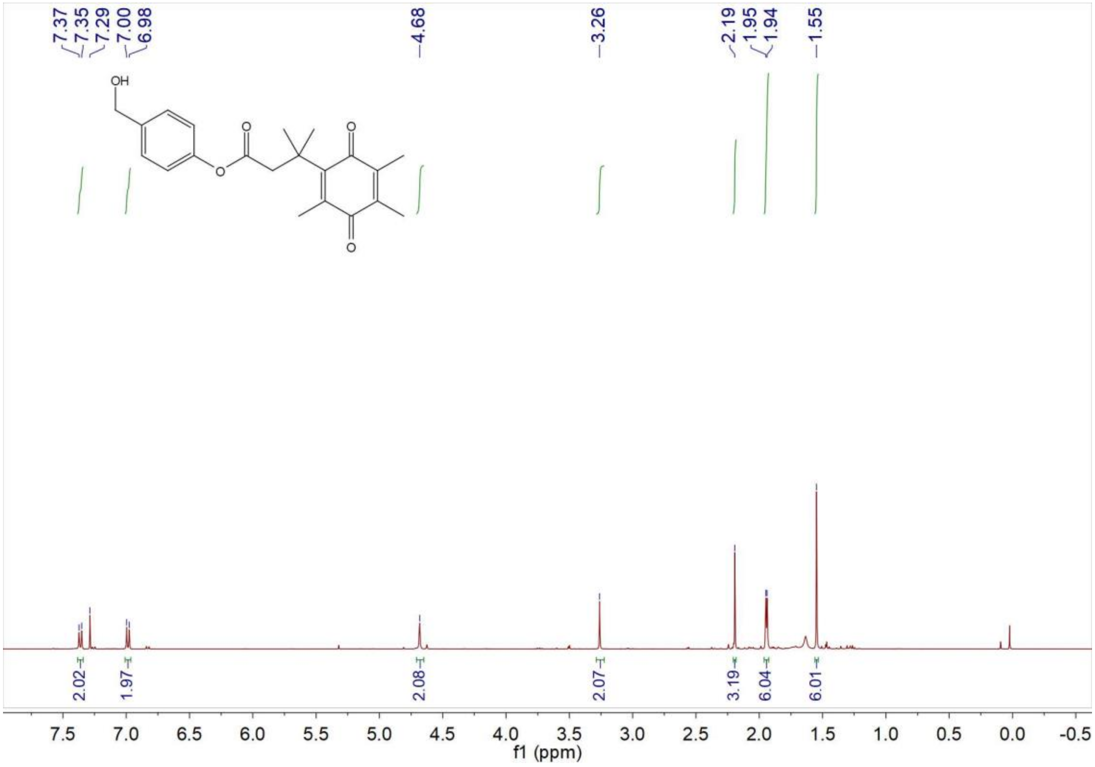

Fig S7. ^{1}H NMR spectrum for compound **7**.

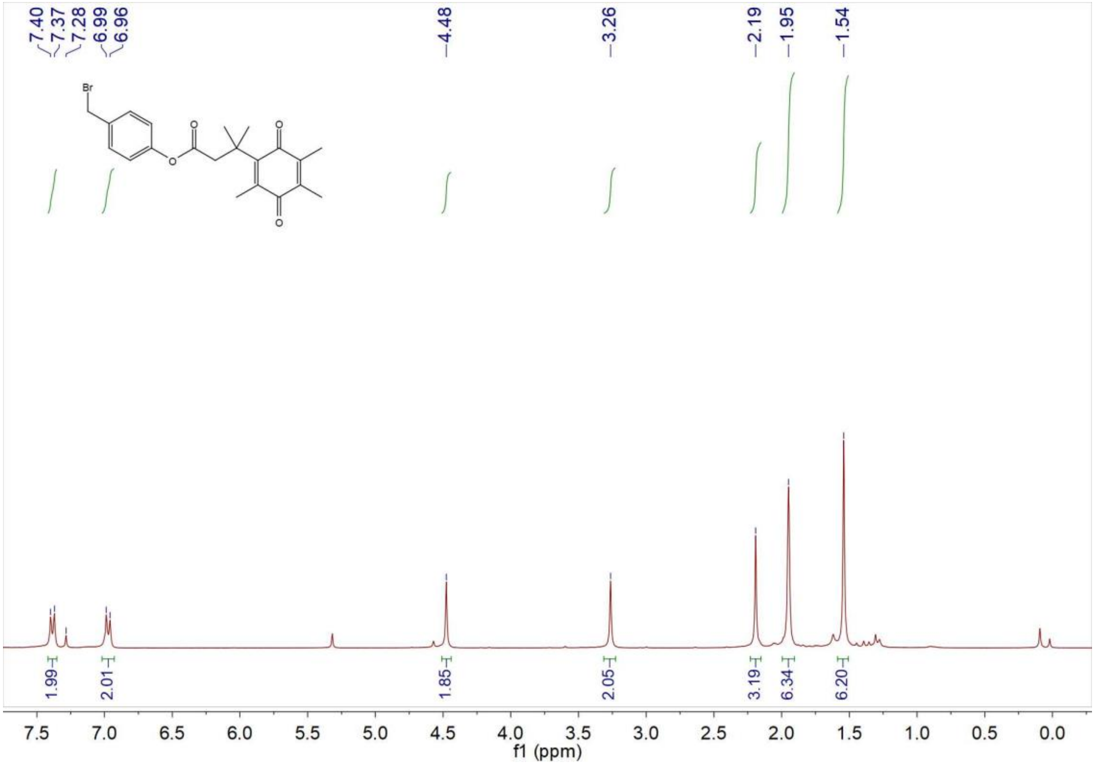

Fig S8. ^{1}H NMR spectrum for compound **8**.

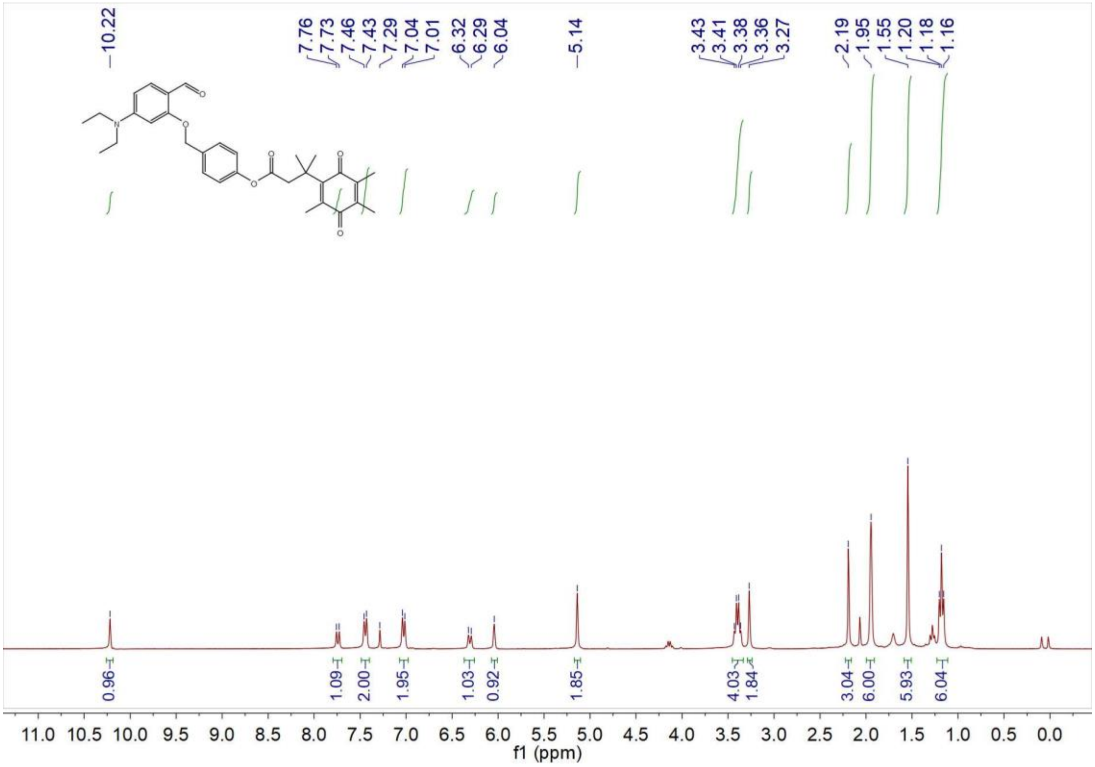

Fig S9. ^{1}H NMR spectrum for compound **9**.

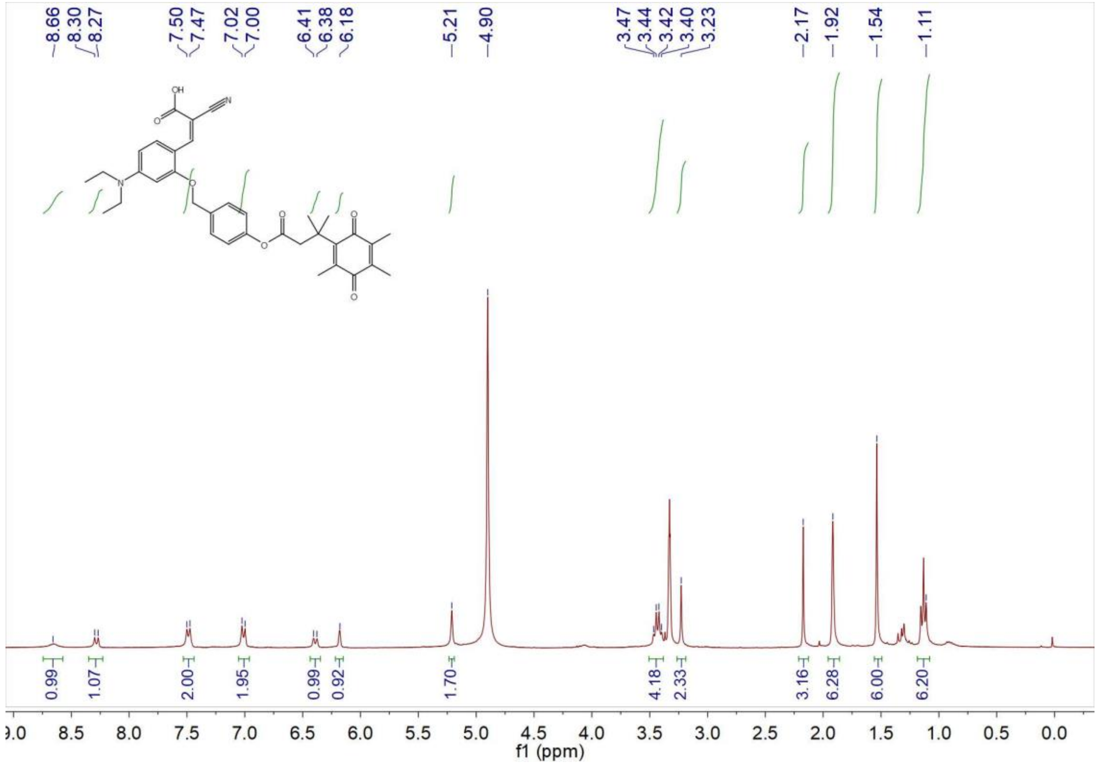

Fig S10. ^{1}H NMR spectrum for probe **1**.

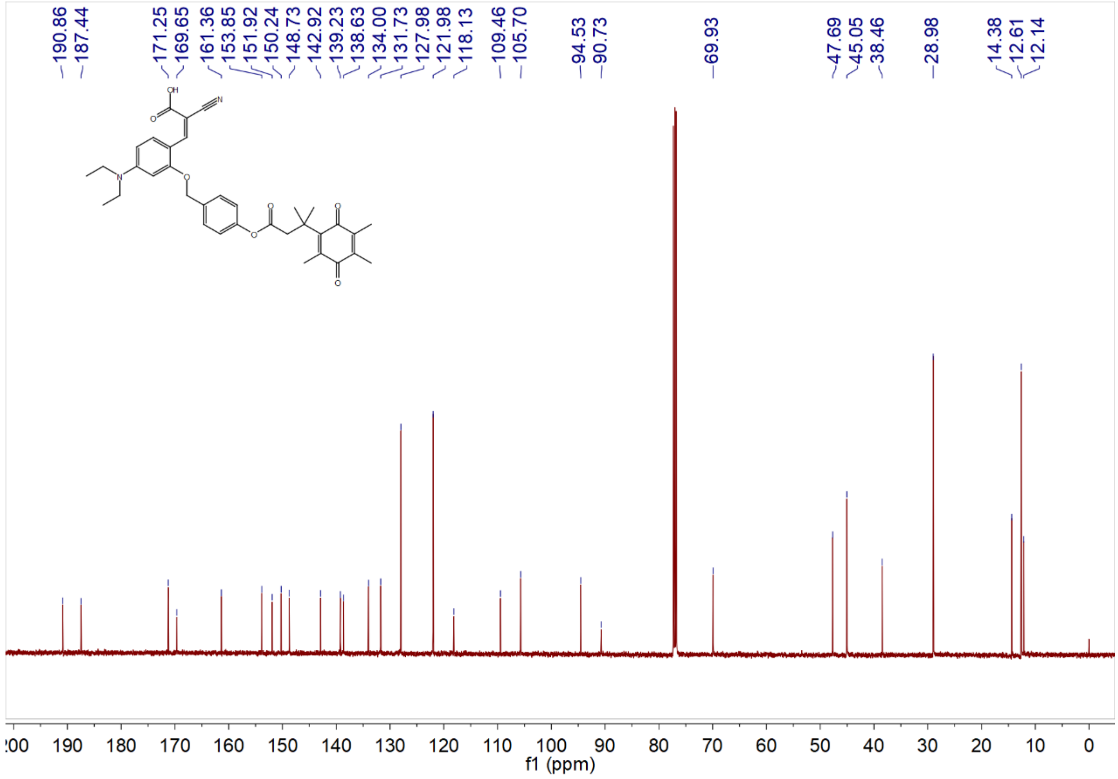

Fig S11. ^{13}C NMR spectrum for probe **1**.

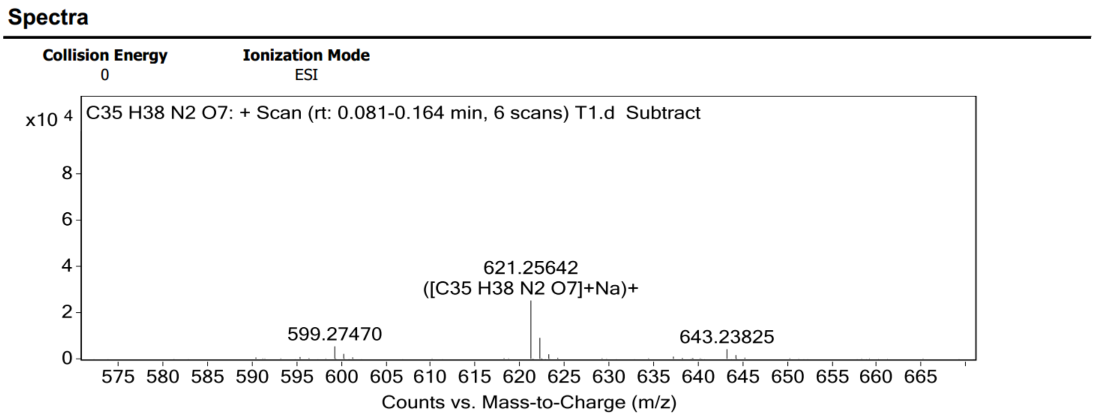

Fig S12. HRMS spectrum for probe **1**.

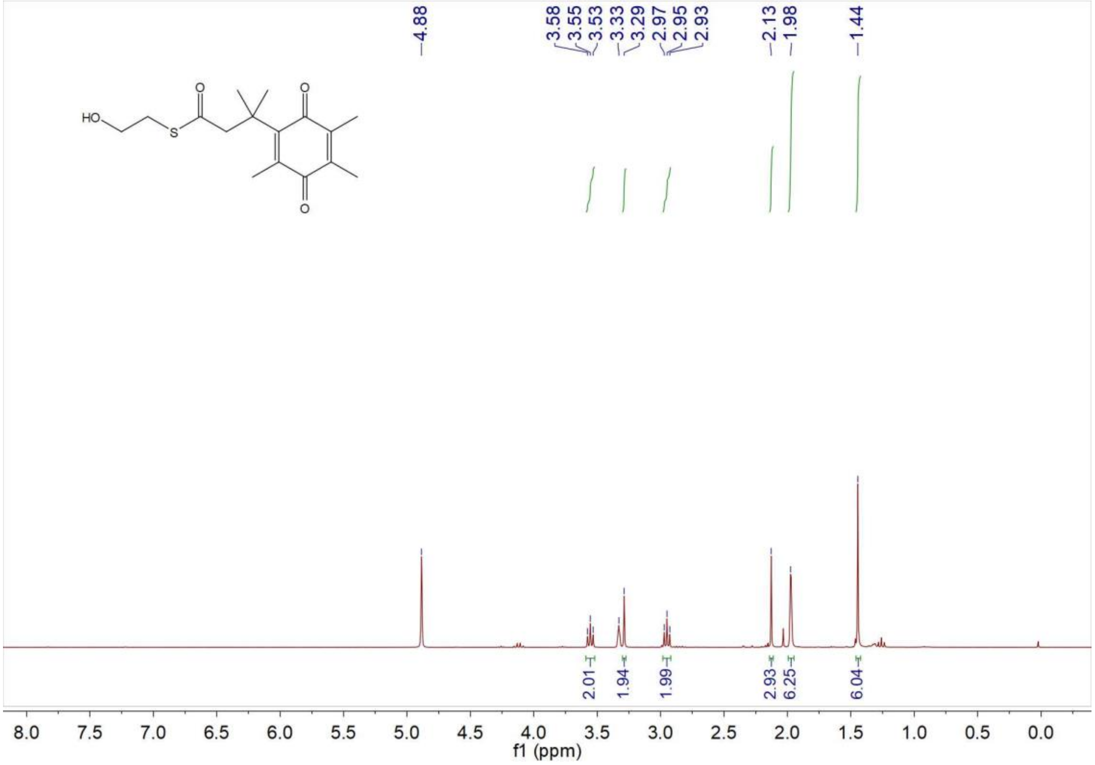

Fig S13. ^{1}H NMR spectrum for compound **10**.

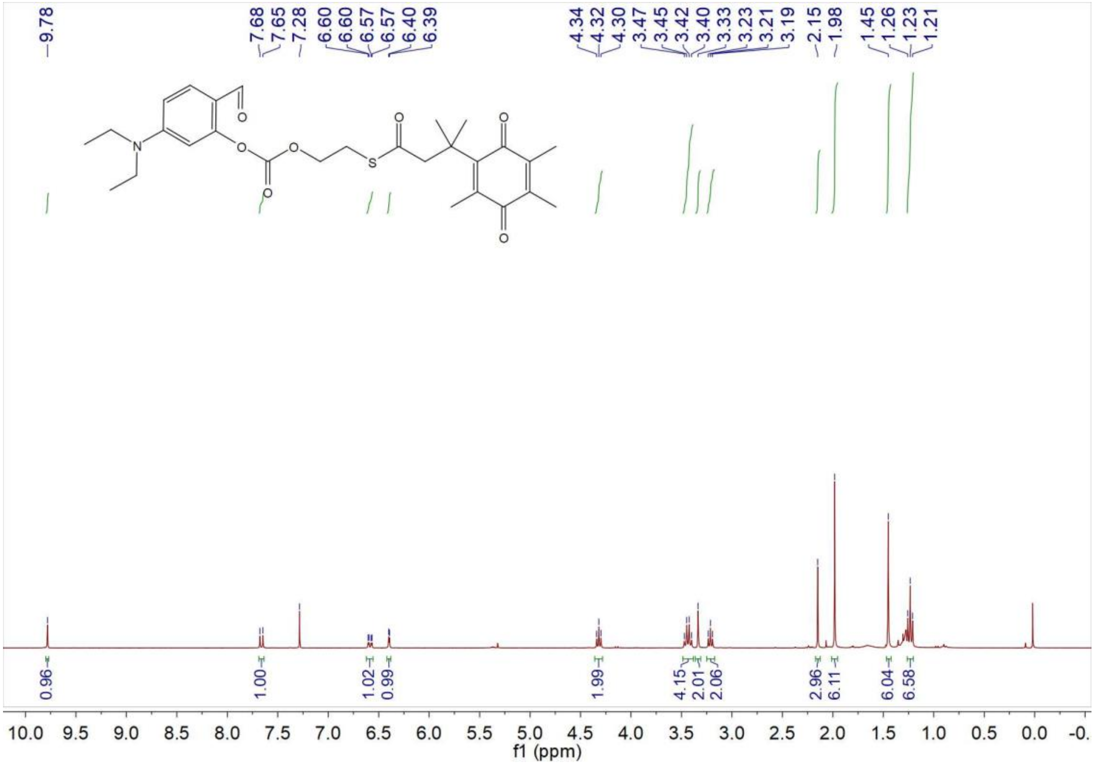

Fig S14. ^{1}H NMR spectrum for compound **11**.

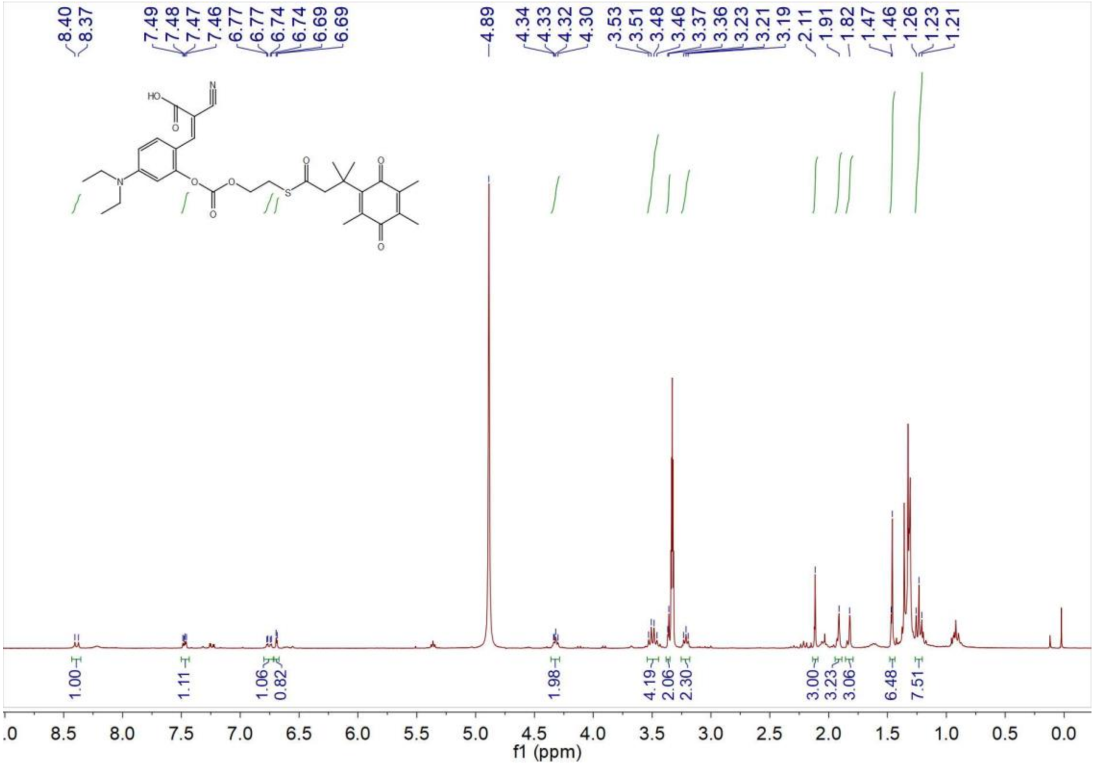

Fig S15. ^{1}H NMR spectrum for probe **2**.

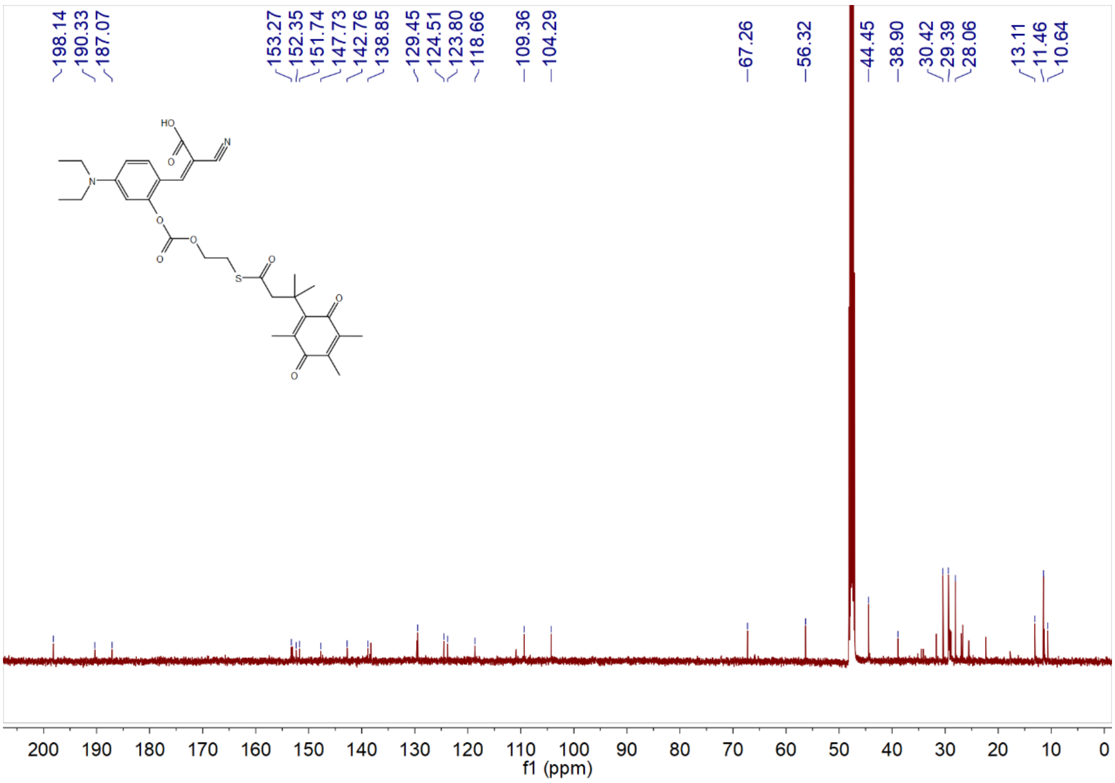

Fig S16. ^{13}C NMR spectrum for probe **2**.

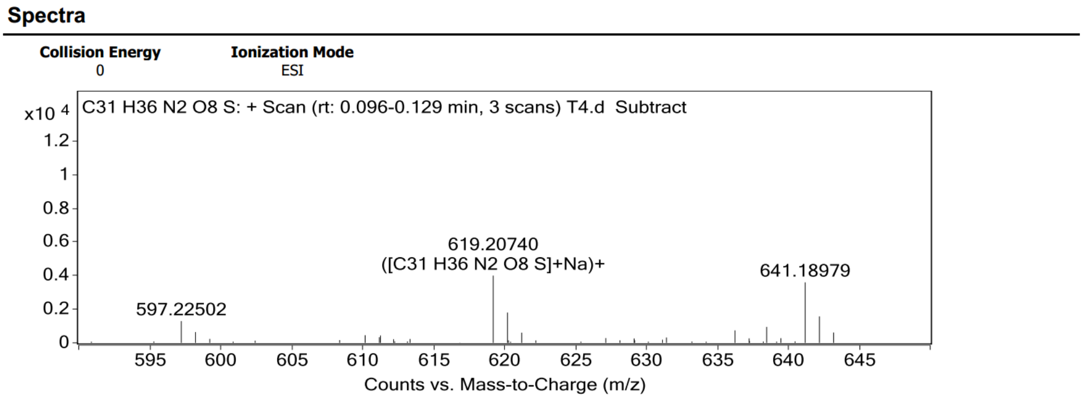

Fig S17. HRMS spectrum for probe **2**.
